# Characterizing cell recruitment into isotropic and anisotropic biomaterials by quantification of spatial density gradients *in vitro*

**DOI:** 10.3389/fbioe.2022.939713

**Published:** 2022-08-05

**Authors:** Martina Tortorici, Erik Brauer, Mario Thiele, Georg N. Duda, Ansgar Petersen

**Affiliations:** ^1^ Julius Wolff Institute, Berlin Institute of Health at Charité—Universitätsmedizin Berlin, Berlin, Germany; ^2^ Berlin-Brandenburg School for Regenerative Therapies, Charité—Universitätsmedizin Berlin, Berlin, Germany; ^3^ BIH Center for Regenerative Therapies, Berlin Institute of Health at Charité—Universitätsmedizin Berlin, Berlin, Germany

**Keywords:** cellular migration, biomaterial architecture, anisotropy, *in situ* tissue engineering, fibroblasts

## Abstract

The success of cell-free *in situ* tissue engineering approaches depends on an appropriate recruitment of autologous cells from neighboring tissues. This identifies cellular migration as a critical parameter for the pre-clinical characterization of biomaterials. Here, we present a new method to quantify both the extent and the spatial anisotropy of cell migration *in vitro*. For this purpose, a cell spheroid is used as a cell source to provide a high number of cells for cellular invasion and, at the same time, to guarantee a controlled and spatially localized contact to the material. Therefore, current limitations of assays based on 2D cell sources can be overcome. We tested the method on three biomaterials that are in clinical use for soft tissue augmentation in maxilla-facial surgery and a substrate used for 3D *in vitro* cell culture. The selected biomaterials were all collagen-derived, but differed in their internal architecture. The analysis of cellular isodensity profiles within the biomaterials allowed the identification of the extent and the preferential directions of migration, as well as their relation to the biomaterials and their specific pore morphologies. The higher cell density within the biomaterials resulting from the here-introduced cell spheroid assay compared to established 2D cell layer assays suggests a better representation of the *in vivo* situation. Consequently, the presented method is proposed to advance the pre-clinical evaluation of cell recruitment into biomaterials, possibly leading to an improved prediction of the regeneration outcome.

## Introduction

Tissue engineering (TE) aims at the restoration, maintenance or improvement of tissue functions (Langer and Vacanti, 1993). This goal is pursued by a number of different strategies, some of which rely on the use of biomaterials to induce specific *in situ* processes in living tissues without additional biological stimuli (Bueno and Glowacki, 2009). Examples can be found, amongst others, in the field of soft tissue augmentation in maxilla-facial surgery. In this context, biomaterials have the potential to overcome the limitations of autologous or allogenic grafts (Toledano et al., 2020). Such limitations may include the need for a second surgical harvesting site and the limited amount of available autologous graft volume (Thoma et al., 2014).

**FIGURE 1:**
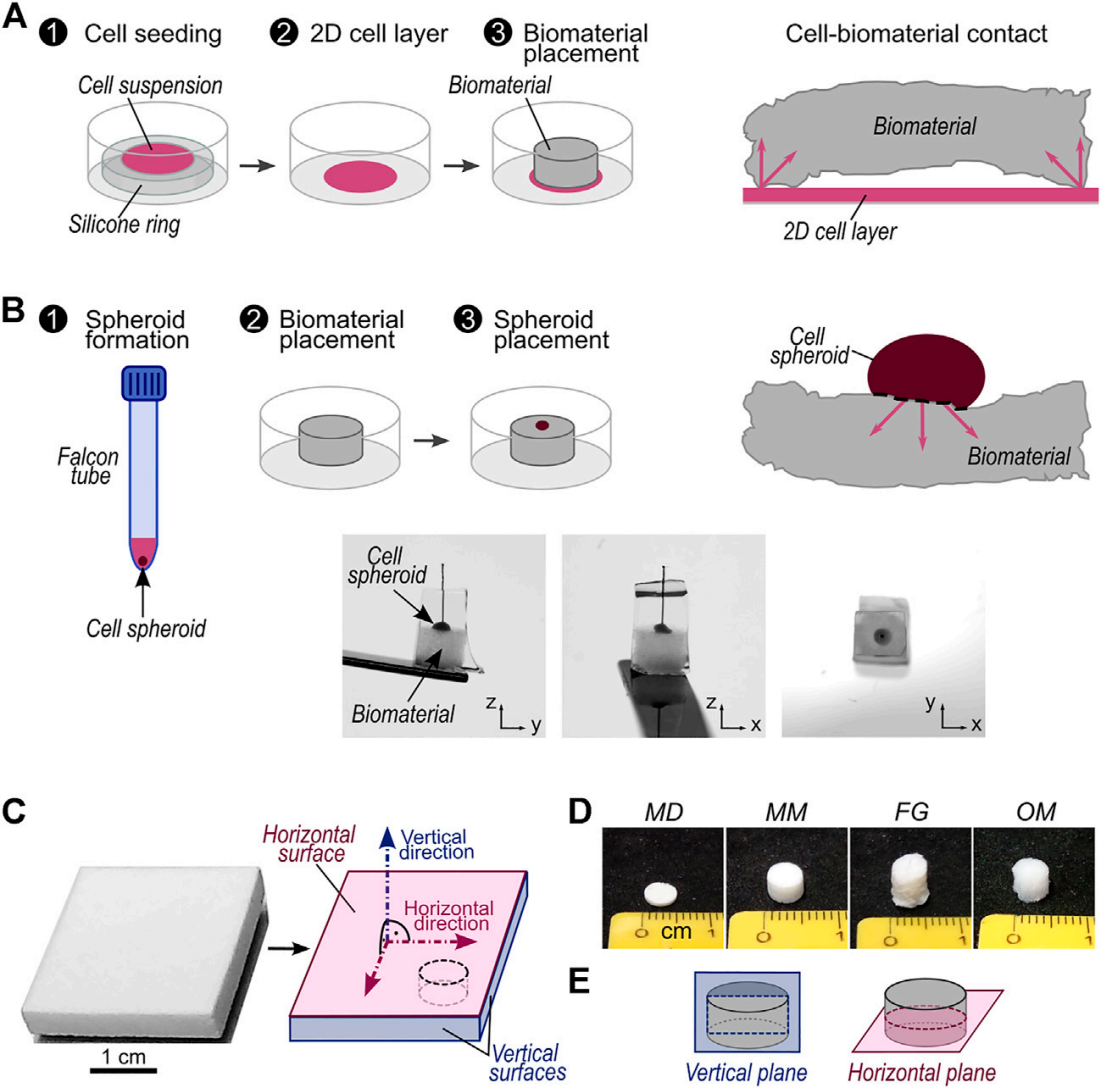
Description of investigated migration assays and biomaterials. **(A)** Schematic workflow of migration assay with 2D cell source and representation of contact between 2D cell source and a biomaterial with non-planar surfaces. The arrows highlight possible directions of cellular migration into the biomaterial; **(B)** top: schematic workflow of migration assay with 3D cell source and representation of contact between 3D cell source and a biomaterial with non-planar surfaces. The arrows highlight possible directions of cellular migration into the biomaterial. Bottom: representative pictures of a cell spheroid placed on a biomaterial sample. The sample is embedded in solidified sucrose/gelatin and a piece of suture material marks the center of the cell spheroid (see **Materials and Methods**); **(C)** Exemplary picture of a biomaterial sheet as delivered by the manufacturer (here Mucomaix) and its schematic representation with indications of the vertical and horizontal surfaces and directions. All vertical surfaces are considered equivalent. The black dashed line marks a representative cutting line for cylindrical samples; **(D)** representative pictures of cylindrical samples of MD, MM, FG, and OM; **(E)** Illustration of vertical and horizontal planes in cylindrical samples.

In cell-free TE-approaches, but also in transplant settings, autologous cell invasion is a key aspect for a fast integration of the implant and determines successive healing. Therefore, the evaluation of cell migration into such biomaterials is essential to establish their competence as pro-regenerative environments, specifically if implanted cell-free. Several two-dimensional (2D) and three-dimensional (3D) approaches are available to study cellular migration *in vitro* (Chung et al., 2010; Wu et al., 2018). The influence of a substrate on the regulation of cellular migration has been extensively studied (Doyle et al., 2013; Janson and Putnam, 2015), e.g., in terms of topography (Berry et al., 2004; Park et al., 2009; Pham et al., 2016; Werner et al., 2017), stiffness (Pelham and Wang, 1997; Lo et al., 2000; Hadjipanayi et al., 2009; Pham et al., 2016), or 3D pore architecture (Wolf et al., 2013; Campbell et al., 2017). Fewer approaches have analyzed the directionality of cell invasion into 3D biomaterial scaffolds that are intended for cell-free clinical applications. So far, cellular migration through a 3D biomaterial has been assessed by seeding cells on one surface and evaluating the number of cells occurring on the opposite surface by either staining (Rampichová et al., 2013) or trypsinization and subsequent flow cytometry measurement (Ravanbakhsh et al., 2020). Although this method can provide valuable insights concerning the barrier function of a biomaterial, it does not allow analyzing how cells invade and distribute within the biomaterial itself. Some studies employ time-lapse imaging to track the movements of life-stained cells inside biomaterials (Harley et al., 2008; Tayalia et al., 2008; Ravanbakhsh et al., 2020). However, such approaches are laborious, not suitable for all types of biomaterials (e.g., not for dense and non-transparent), require specialized equipment and capture only a low number of cells. Furthermore, phototoxicity and photobleaching may influence cellular migration behavior (Wu et al., 2018). In other reported methods, the biomaterial is cultured in contact with a cell source and the cellular distribution is analyzed by staining (Mandal and Kundu, 2009; Murphy et al., 2010; Petersen et al., 2018; Huang et al., 2021). We recently employed such an approach in our lab to assess cell invasion into a biomaterial from a confluent 2D monolayer (Petersen et al., 2018) (Figure 1A). However, this method only provides a limited amount of cells to invade the biomaterial, the cell density strongly decreases as cells distribute within the 3D environment, and its reliability depends on a perfect contact of the biomaterial with the cell monolayer. Lastly, such an assay is not capable of capturing a possible anisotropy of cell migration (resulting from the architecture of the biomaterial) within one experiment.

Here, we propose a new approach that overcomes these limitations and allows the assessment of the extent and directionality of cell migration into 3D biomaterials within a single experiment. Furthermore, the presented method does not require specialized equipment in addition to standard cell-culture tools. We tested three clinically applied porcine-derived collagen-based biomaterials for soft tissue augmentation: Fibro-Gide^®^ (FG) (Zeltner et al., 2017), Mucoderm^®^ (MD) (Stricker et al., 2014), and Mucomaix™ (MM) (Wessing and Vasilic, 2014). Primary human dermal fibroblasts (hFBs) were employed here due to their relevance in respect to the clinical application of the biomaterials. To prove the ability of the novel assay to evaluate migration in anisotropic biomaterials, a well-established 3D cell culture substrate with anisotropic morphology, i.e., Optimaix™ (OM) (Petersen et al., 2018; Brauer et al., 2019; Herrera et al., 2019; Schreivogel et al., 2019), was analyzed additionally. To be able to explain the differences in cell migration observed for the individual biomaterials, their material orientation, pore size, and mechanical stiffness were characterized in detail. We expect that the new 3D spheroid assay will bring further understanding of the cell-biomaterial interactions, thereby improving tissue engineering approaches.

## Materials and Methods

### Cell culture

Primary human fibroblasts (hFBs) were isolated from skin biopsy by outgrowth culture. Cell culture was performed in expansion medium composed of Dulbecco’s modified Eagle’s medium (DMEM, Thermo Fischer), 10% fetal bovine serum (FBS, Biochrom AG), 1% penicillin/streptomycin (P/S, Biochrom AG), and 1% non-essential amino acids (NEA, Biochrom AG). Trypsinization (PAA Laboratories GmbH) was performed when cells reached 80% confluency. Experiments were performed with cells in passages 3 to 8 with no difference in mean passage number for the investigated biomaterials.

### Sample preparation

Commercially available samples of Fibro-Gide^®^ (FG, Geistlich Pharma AG, Switzerland), Mucoderm^®^ (MD, Botiss Biomaterials GmbH, Germany), and Mucomaix™ (MM, Matricel GmbH, Germany) were obtained from the manufacturers as sterilized products intended for clinical use. Similarly, commercially available samples of Optimaix™ (OM, Matricel GmbH, Germany), serving as a well-characterized reference biomaterial (Petersen et al., 2018; Brauer et al., 2019; Herrera et al., 2019), were obtained from the manufacturer as sterilized products intended for *in vitro* cell culture. All materials were used to compare a previously developed migration assay based on a 2D cell layer adhered to tissue culture plastic as a cell source (Figure 1A) with the here-introduced assay that employs a 3D cell spheroid directly attached to the material (Figure 1B). Each investigated biomaterial was received as a sheet on which horizontal and vertical surfaces were defined as illustrated (Figure 1C). Moreover, vertical and horizontal directions were defined as the normal to the horizontal and vertical surfaces, respectively. All vertical surfaces were considered equivalent. To perform the experiments and characterizations described in the following sections, specimens were cut from the commercial sheets of biomaterials, maintaining the definitions established on the as-received products. In the case of cylindrical samples, of which representative pictures for each biomaterial are shown in Figure 1D, horizontal and vertical planes were additionally defined as illustrated in Figure 1E.

### Scanning electron microscopy

Biomaterial morphology was evaluated qualitatively by scanning electron microscopy (SEM, JCM-6000, Jeol). Samples underwent gold sputtering for 30 s at 8 Pa pressure and 40 mA electric current, followed by secondary electrons imaging in high vacuum.

### Pore size, collagen wall distance, and biomaterial anisotropy evaluation

Pore size and biomaterial anisotropy were evaluated by visualizing FBS-derived fibronectin adsorbed on the collagen walls together with the collagen signal of the biomaterial detected by second harmonic (SH) generation, as previously described (Herrera et al., 2019). Biomaterials (n = 3) were incubated overnight at 37°C in FBS and 6.7% P/S. Samples were washed with phosphate buffered saline (PBS, Life Technologies Limited), fixed with 4% paraformaldehyde solution and stained for fibronectin (primary antibody: anti-fibronectin antibody, #ab23750, Abcam plc; secondary antibody: Alexa Fluor™ 555 donkey-anti-rabbit, Thermo Fisher). Confocal imaging was performed using a 25× water immersion lens (Leica SP5 II confocal laser scanning microscope, Leica Microsystems GmbH). Second harmonic imaging was performed using a Mai Tai HP tunable IR laser (Newport Spectra Physics) with excitation at 910 nm and detection at 450–460 nm. To evaluate biomaterial anisotropy, the vertical plane of the samples was imaged (defined in Figure 1C), recording image stacks with a voxel size of 1.2 µm × 1.2 µm × 5 µm (5 µm in-depth spacing) and a stack depth of 50 µm. To evaluate the pore size, the horizontal plane of the samples was imaged, recording image stacks in two separate regions of the samples with an area of 2,480 × 2,480 μm^2^, a voxel size of 1.2 µm × 1.2 µm × 2 μm, and a stack depth of 50 µm. To obtain images with a clear fibronectin signal also in the bulk of the samples, a linear laser power compensation for this signal was employed. In both evaluations, areas of the MD samples smaller than the recorded images were analyzed to exclude regions where the biomaterial was out of plane. For all the recorded image stacks, the SH and fibronectin signals were combined and subsequently binarized.

Biomaterial anisotropy was evaluated using the OrientationJ plugin in ImageJ (Fonck et al., 2009; Schindelin et al., 2012). Specifically, the “Distribution” function of OrientationJ was employed on five consecutive planes of the confocal image stacks. The resulting orientation distributions were normalized to the sum of all the values over 360°. Results are given as mean values of the normalized values (in percentage) ± standard deviation.

The pore size was evaluated on 3D confocal stacks using the BoneJ plugin for ImageJ (Doube, 2020). The pore size is given as the mean of the measured sphere diameters ±standard deviation. The distribution of the pore diameter was obtained by extracting and smoothening the pore diameter histogram (Savitzky-Golay method with 20 points of window and a polynomial order of 5) in Origin 2019b (OriginLab Corporation).

In MM and OM, the distance between parallel walls was additionally measured manually in ImageJ from the confocal images. Results are given as average wall distance ±standard deviation.

### Mechanical compression tests

Monoaxial compression tests were performed on a BOSE ElectroForce Test Bench (TA Instruments ElectroForce System Group) by applying a 1 mm displacement to the samples and recording their reaction force with a 0.49 N-load cell. The low thickness of MD samples required a specific setup of the compression test, during which a compression of 10% of the specific sample height was applied and the reaction force was measured with a 9.8 N-load cell. Biomaterials were placed in PBS for at least 1 h prior to testing, as a rehydration-dependency of mechanical properties has been reported for MD (Kasaj et al., 2015). During the compression tests, samples were completely immersed in PBS. Measurements were converted into stress and strain. Compressive elastic moduli (*E*) were calculated from the linear trait of the stress-strain curves. Specimens (n = 6) were compressed along the vertical and the horizontal directions (see Figure 1C) to measure *E*_*V*_ and *E*_*H*_, respectively.

### 2D_V_ and 2D_H_ layer migration assays

Migration assays from a 2D cell source (2D layer assays) were performed as previously described (Petersen et al., 2018) (Figure 1A). Briefly, a custom-made silicon ring (inner diameter of 7 mm) was placed in a 12-well plate (Corning Incorporated) and 0.2 × 10^6^ cells were pipetted in the middle of the ring and incubated at 37°C and 5% CO_2_ for 1 h. After removing the silicon ring and performing two washings with PBS to eliminate non-adherent cells, biomaterial samples were first wetted in hFBs expansion medium and then placed onto the 2D cell layer. Cellular migration was studied either along the vertical (2D_V_ layer assay) or the horizontal (2D_H_ layer assay) directions (see Figure 1C) by placing the horizontal or vertical surfaces, respectively, in contact with the cell layer. Custom-made silicon holders were used to hold MD samples in place for the 2D_H_ layer assay due to their low thickness (membrane-like biomaterial). After placing the samples, expansion medium was added until almost reaching the top surface of the samples, followed by a 24-h incubation at 37°C and 5% CO_2_. Samples were transferred to a new 12-well plate with fresh expansion medium. To reduce the potential influence of gravity on the outcome, samples were placed sideways (seeded surface vertically) to make cell migrate horizontally. The only exception were the MD samples of the 2D_V_ layer assay, which were too thin to be held in such a configuration. Cell culture was carried on for 5 days, with medium exchange at day 3. Subsequently, samples were fixed with 4% PFA and processed as later described.

### 3D spheroid migration assay

To prepare the spheroids, a suspension containing 0.5 × 10^6^ cells was centrifuged in 15-ml Falcon tubes at 1,500 rpm for 1 min and subsequently incubated at 37°C and 5% CO_2_ for 24 h (Figure 1B). The lids of the falcon tubes were not fully closed to enable gas exchange. To keep samples upright, a 24-well plate was prepared with custom-made silicon holders, which were filled with expansion medium. Samples were placed in the middle of the holders and the cell spheroid was placed on top of the sample using a trimmed 1-ml tip. Samples were incubated at 37°C and 5% CO_2_ for 3 h and successively transferred to a 12-well plate with fresh expansion medium. Similarly to the 2D layer migration assays, the potential influence of gravity was reduced by culturing the samples with the migration direction parallel to the bottom of the well plate, except MD samples. Cell culture was carried on for 5 days with medium exchange at day 3.

### Fixation and sample processing

All samples were fixed with 4% paraformaldehyde (PFA), followed by a 1 h immersion in a 25 mM ammonium chloride solution in PBS. Samples were further washed in PBS and incubated in a solution of 5% sucrose/5% gelatin in PBS.

Samples of the 2D layer assay were placed in a custom cutting mold filled with sucrose/gelatin solution, which was solidified by a 1 h incubation at 4°C. Next, samples were cut in two halves with a scalpel and washed in PBS until complete removal of the residual sucrose/gelatin. Finally, samples were embedded in TissueTek^®^ (Sakura FinetekEurope B.V.) and the imaging surface was trimmed using a cryomicrotome (Leica CM3550 S). TissueTek^®^ was removed by PBS washings.

Samples of the 3D spheroid migration assay were transferred to a 12-well plate. Samples were placed with the migration direction parallel to the bottom of the wells and with the non-cell-seeded bottom surface in contact with the side of the well. Wells were filled with sucrose/gelatin solution until samples were completely immersed and solidified for 1 h at 4°C. Excess sucrose/gelatin was removed from around individual samples, leaving 3–4 mm of extra-sucrose/gelatin on the spheroid side. A piece of suture material (Ethicon Prolene 5–0, Ethicon LLC) was stuck into the extra-sucrose/gelatin, pointing the middle of the cell spheroid to indicate its position (Supplementary Figure S1). Sucrose/gelatin cubes containing samples and suture material were embedded in TissueTek^®^ and cut at the cryomicrotome until reaching the suture material, i.e., the middle of the cell spheroid. TissueTek^®^ and sucrose/gelatin were removed by repeated washings in PBS.

### Analysis of cell migration distance

Samples from 3D spheroid and 2D_V_ and 2D_H_ layer migration assays were stained for actin filaments (F-actin, Alexa fluor 488 Phalloidin, #A12379, Thermo Fisher or Phalloidin Atto 633, #68825, Sigma) and cell nuclei (DAPI, #D3571, Thermo Fisher or SYTOX Green, #S7020, Invitrogen) before imaging with confocal microscopy. Image stacks were recorded with a voxel size of 1.2 µm × 1.2 µm × 5 µm and a stack depth of 75 µm.

**FIGURE 2:**
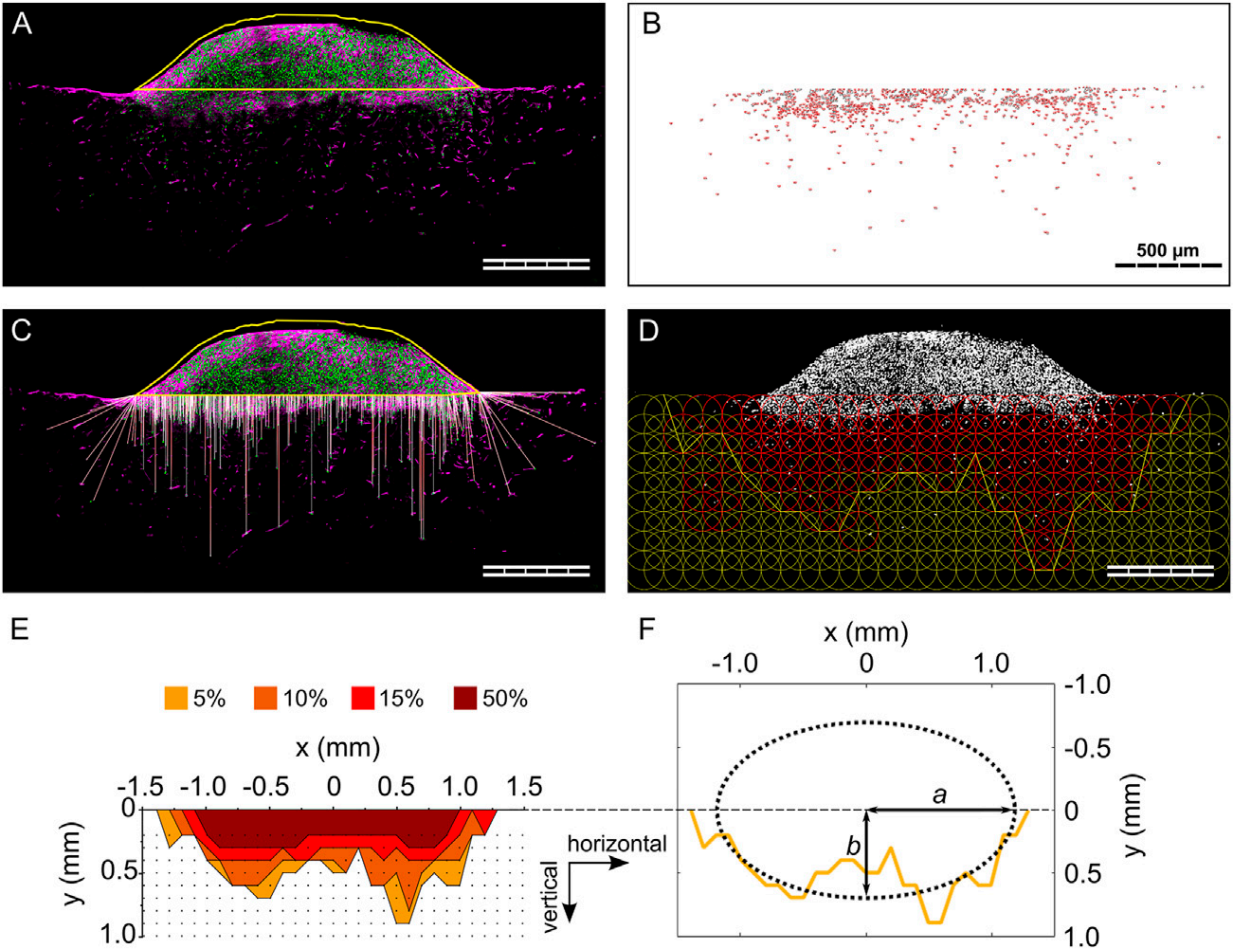
Individual steps of the analysis procedure for the 3D spheroid migration assay. **(A)** Outline of cell spheroid; **(B)** cell nuclei count within the biomaterial; **(C)** visualization of the calculated 3D migration distances (*d*_*3D*_) for each cell; **(D)** visualization of the grid of ROIs and indication of the 5% isodensity profile. Cell nuclei are visualized in gray. ROIs containing a cell density equal to or higher than the specified threshold are colored in red; **(E)** overlay of 5%, 10%, 15%, and 50% isodensity profiles in the relative coordinate system. The black dots correspond to the points of the circular ROIs in the grid according to **(D)**; **(F)** elliptic fit exemplary shown for the 5% isodensity profile. The semi-major and semi-minor axes (*a* and *b*, respectively) are marked. All images refer to the same representative sample (MM) from one 3D spheroid migration experiment. Scale bars are 500 µm. **(A and C)** show cell nuclei in green and F-actin in magenta.

The cell migration distance was analyzed from maximum projections of four consecutive focal layers. For the 3D spheroid assay, the spheroid region, defined as the part of the spheroid above the biomaterial surface, was manually outlined and excluded from the analysis area (Figure 2A). A threshold was set manually to binarize the images. Individual cell nuclei were automatically identified based on size and circularity using the “Analyze particles” function of ImageJ and their coordinates were recorded (Figure 2B). The minimum distance of each nucleus from the spheroid outline was calculated and defined as migration distance (*d*_*3D*_, Figure 2C). The analysis steps, which were performed semi-automatically by means of an ad-hoc ImageJ macro, are illustrated in Supplementary Figure S2. For the 2D_V_ and 2D_H_ layer assays, the same procedure was performed. However, in this case, the migration distances (*d*_*2DV*_ and *d*_*2DH*_, respectively) were calculated by outlining the whole surface that was in contact with the confluent cell layer.

### Analysis of migration profile and definition of isodensity ellipses

The anisotropy of cellular migration in the 3D spheroid assay was analyzed using ImageJ and Matlab (MathWorks). Spheroid outline, cell nuclei positions, and nuclei count were taken from migration distance analyses. To analyze the local cell density, a circular region of interest (ROI, diameter of 200 µm) was defined and overlaid in a repetitive, grid-like configuration. Individual ROIs did overlap by 50% of their diameter to smoothen local differences in cell density in individual pores. Next, the local cell density based on particle count was calculated for each ROI (Figure 2D), identifying ROIs containing a cell density equal to or higher than four thresholds of 50, 95, 150, and 475 cells/mm^2^. These thresholds corresponded to 5%, 10%, 15%, and 50% of a reference cell density of 950 cells/mm^2^. The reference cell density was identified in preliminary evaluations as a representative number of a quarter of the highest cell density found close to the spheroid. For each column of the grid, the ROI most distant from the biomaterial surface and containing a cell density equal to or higher than the specified cell density threshold (5%, 10%, 15% or 50%) was identified (= threshold ROI). To reduce scattering resulting from local variations of cell densities, an additional criterion was applied in the definition of the threshold ROIs, requiring the next two ROIs of the column (more distant from the biomaterial surface) to both have a cell density lower than the threshold. That point on the perimeter of the threshold ROI that was most distant from the biomaterial surface was selected and the according points of all columns were connected by a polygonal line and defined as isodensity profiles (Figure 2D). The analysis steps, also in this case performed with an ad-hoc ImageJ macro, are illustrated in Supplementary Figure S3.

The origin of the coordinate system containing the isodensity profiles was chosen to be in the center of the profiles horizontally and to be positioned on the cell spheroid–biomaterial interface vertically (Figure 2E). The zero-position of the horizontal coordinate was calculated as the average of the coordinates dividing in half the area underlying the 5%, 10%, and 15% isodensity profiles.

The anisotropy in cellular migration was evaluated by fitting the isodensity profiles with ellipses, which were called isodensity ellipses. To do so, a Matlab script incorporating a previously established method was used (Halir and Flusser, 1998) (Figure 2F). The fitting resulted in the six algebraic parameters (here called *k*_*1*_ to *k*_*6*_) that describe an ellipse according to Eq. 1:

$$k_{1}x^{2}+k_{2}xy+k_{3}y^{2}+k_{4}x+k_{5}y+k_{6}=0$$ (1)

Thus, the computation of semi-major (*a*) and semi-minor (*b*) axes was performed as described in Eq. 2, selecting *b* < *a* by definition:

$$a,\;b=\;\sqrt{\frac{2\left(k_{1}k_{5}^{2}+k_{3}k_{4}^{2}+k_{6}k_{2}^{2}-2k_{2}k_{4}k_{5}-k_{1}k_{3}k_{6}\right)}{\left(k_{2}^{2}-k_{1}k_{3}\right)\left[\pm \sqrt{{\left(k_{1}-k_{3}\right)}^{2}+4k_{2}^{2}}-\left(k_{1}+k_{3}\right)\right]}}$$ (2)

The orientation of *a* and *b* along the vertical or horizontal directions was determined by comparison with the isodensity profiles. The ellipses resulting from the fitting procedure were averaged between samples, obtaining the mean 5%, 10%, 15%, and 50% isodensity ellipses for MD, MM, FG, and OM.

To calculate the horizontal extension of the migration profile, the spheroid radius *P* (describing the horizontal width of the cell source) had to be subtracted from the semi-major axis *a* of the ellipses. A spheroid-size-exclusion procedure was proposed as described in the following paragraph. Such a procedure was first established on a reference biomaterial to set the required parameters. Then, it was applied to the other investigated biomaterials. The results of the proposed procedure were compared to the ratio *R* between migration distances in the well-established 2D_V_ and 2D_H_ layer assays, as defined by Eq. 3:

$$R=\frac{d_{2DV}}{d_{2DH}}$$ (3)

OM was chosen as reference biomaterial because of its previously performed extensive characterization in terms of material properties (Brauer et al., 2019; Herrera et al., 2019) and cellular migration (Petersen et al., 2018; Schreivogel et al., 2019). For each isodensity ellipse (*i*) of 5%, 10%, and 15%, the spheroid radius *P*_OM,i_ of the reference biomaterial OM was calculated according to Eq. 4 as the value to subtract from the semi-major axis *a* to obtain a ratio *b*_*OM,i*_/(*a*_*OM,i*_ – *P*_*OM,i*_) expressing an anisotropy of migration equal to the ratio *R* calculated for OM (*R*_*OM*_):

$$\frac{b_{OM,i}}{a_{OM,i}-P_{OM,i}}=R_{OM}\;\to \;P_{OM,i}=\;a_{OM,i}-\left(b_{OM,i}\cdot \frac{1}{R_{OM}}\right)$$ (4)

The 50% isodensity profile was excluded, as it was not representative for the general migration profile for any of the investigated biomaterials.

The average of the so calculated spheroid radius for the 5, 10, and 15% isodensity ellipses of OM (*P*_*OM,avg*_) was subtracted from the semi-major axes calculated for the 5%, 10%, and 15% isodensity ellipses of the other investigated biomaterials, i.e., MD, MM, and FG. Anisotropy in migration in the 3D spheroid assay was evaluated as the ratio R* between the semi-minor axis and the resulting difference, as indicated by Eq. 5:

$$R^{*}=\frac{b}{a-P_{OM,avg}}=\frac{b}{a^{\prime }}$$ (5)

Additionally, the length of the cell spheroid–biomaterial contact was manually measured for each biomaterial from the confocal images of the 3D spheroid assay to justify the use of the same spheroid width (*P*_*OM,avg*_) for all materials (shown in Supplementay Figure S7). The information was also important to verify that the differences between 2D layer and 3D spheroid assay described later for MM (Figure 5D) were not a consequence of an over-estimation of the spheroid contact length for this particular material.

### Analysis of cell density at the cell source-biomaterial interface

Cellular density was measured near the surface that was in contact with the cell source in 2D_V_ and 2D_H_ layer and in 3D spheroid migration assays. For 3D spheroid assays, a ROI with constant height of 200 µm that followed the profile of the biomaterial surface was defined under the cell spheroid (results shown in Figure 6). For 2D layer assays, the analysis region had a height of 200 µm and a length equal to the average contact between cell spheroid and biomaterials. Cellular density was calculated based on particle count within the respective ROIs normalized to the ROI surface and is given as average ±standard deviation. As cells did not extensively populate the bulk of MD, this biomaterial was excluded from the analysis.

### Evaluation of migration profiles by computational simulations

Diffusion profiles in presence of isotropic and anisotropic diffusion coefficients were obtained by solving a mass diffusion problem in a finite element (FE) solver (Abaqus, Dassault Systèmes). The biomaterial samples analyzed experimentally were computationally represented by a 2D square (5 × 5 mm^2^), which was meshed with 2,500 quadratic elements type DC2D4 (for a total of 2,601 nodes). To model the cell spheroid, a line source comprising the surface nodes of 18 elements, corresponding to the average cell spheroid–biomaterial contact, was defined in the middle of one side of the square and was assigned a concentration of 100%. In the FE model, the vertical and horizontal directions were defined as the normal and parallel directions, respectively, to the line source and belonging to the plane of the square. Three mass diffusion problems were investigated: I) vertical diffusion coefficient 2-fold higher than the horizontal one; II) equal vertical and horizontal diffusion coefficients; and III) horizontal diffusion coefficient 2-fold higher than the vertical one. The results experimentally obtained for the reference biomaterial, i.e., OM, in the reference migration assay, i.e., the 2D layer assay, were used to define representative diffusion parameters for the simulations. The average migration distance along the vertical direction of OM (500 µm) over the experimentally investigated time (5 days) was considered the result of a constant migration speed, resulting in a calculated migration speed value of 1.16 × 10^–6^ mm/s. The higher (*HD*) and lower (*LD*) diffusion coefficients in the computational simulations were approximated by the calculated migration speed and half of its value, respectively. Thus, the three investigated simulations were performed with the following vertical: horizontal diffusion coefficients: I) *HD*: *LD*; II) *LD*: *LD*; and III) *LD*: *HD*. Moreover, a solubility and a concentration of 1 were assigned in the definition of the material properties. A diffusion step of 432,000 s, corresponding to 5 days, was performed and the resulting 5%, 10%, 15%, and 50% concentration profiles were evaluated.

### Statistical analysis

Statistical significance between data sets was assessed in Origin 2019b using a two-sided Mann-Whitney-U test with Bonferroni correction for multiple comparisons. Significance levels between the median of a data set and a fixed value were evaluated with a one-sample Wilcoxon Signed Rank test. A value of *p* < 0.05 was considered as statistically significant and was indicated by an asterisk (*), whereas values of *p* ≥ 0.05 were indicated as non-significant (n.s.).

## Results

### Qualitative morphological characterization

Four porcine collagen-derived biomaterials were employed to compare a well-established migration assay with 2D cell source (2D layer assay, Figure 1A) and a novel assay with 3D cell source (3D spheroid assay, Figure 1B). For each commercial sheet of biomaterial vertical and horizontal surfaces and directions were defined to evaluate the cellular migration guided by isotropic and anisotropic materials (Figure 1C). The pore morphology of MD, MM, and FG (exemplary pictures of the materials in Figure 1D) was qualitatively evaluated via scanning electron microscopy (SEM) on vertical and horizontal cuts through the material (according to Figure 1E). Optimaix, an *in vitro* cell culture substrate with highly anisotropic pore architecture, was used as a reference material. The different types of materials were chosen to evaluate the here-suggested 3D spheroid assay (depicted in Figure 2).

**FIGURE 3:**
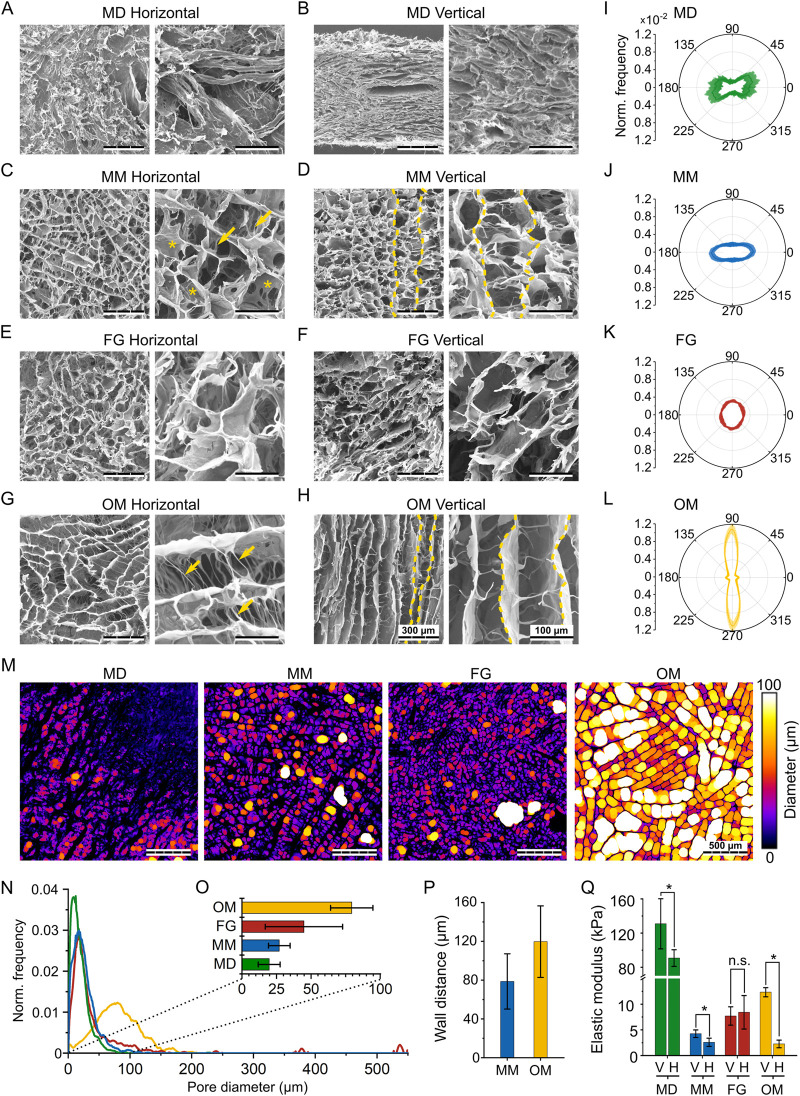
Structural and mechanical characterization of the investigated biomaterials. **(A,C,E,G)** SEM images of the horizontal plane of MD, MM, FG, and OM, respectively; **(B,D,F,H)** SEM images of the vertical plane of MD, MM, FG, and OM, respectively. For each material, images on the right show the internal structure at a greater magnification. Scale bars are 300 µm (overview) and 100 µm (magnification). In **(D and H)**, yellow dashed lines mark representative vertical walls. In the higher magnification images of **(C and G)**, arrows and asterisks highlight fiber- and sail-like horizontal bridges, respectively; **(I–L)** normalized distribution of orientation in MD, MM, FG, and OM. Orientations of 0° and 90° indicate the horizontal and vertical directions, respectively. Results are shown as mean values (solid line) ± standard deviation (shaded area); **(M)** Representative images visualizing the automated pore size measurement in the investigated biomaterials. Colors indicate the diameters of the spheres that were fitted into the pores, while black areas represent the material. Scale bar is 500 μm; **(N)** Frequency distribution of the automatically measured pore diameters. Results were normalized to the sum of all measured diameters and the curve was smoothened for better representation; **(O)** mean pore size ±standard deviation derived from the automated measurements; **(P)** average wall distance ±standard deviation in MM and OM obtained from manual measurement; **(Q)** compressive elastic modulus (mean ± standard deviation) along the vertical (V) and horizontal (H) direction of the investigated biomaterials.

MD was composed of bundles of fibers, which formed a compact surface in the horizontal plane (Figure 3A) and appeared as layers in the vertical plane (Figure 3B). On the contrary, the other three biomaterials showed interconnected macro-pores, both in the vertical and in the horizontal planes (Figures 3C–H). The structure of both MM and OM was composed of domains of parallel walls with random orientations in the horizontal plane (Figures 3C,G, respectively) and parallel vertically oriented collagen walls in the vertical plane (see yellow dashed lines in Figures 3D,H, respectively). Despite similarities in the architecture of MM and OM, the difference between vertical and horizontal planes was much less apparent for MM than for OM. This was the result of a different distribution of the material in between the walls. While in OM exclusively fiber-like connections were found, these “bridges” were heterogeneous in morphology in MM, appearing as fibers but also as sail-like membranes (see higher magnification pictures in Figures 3C,G for MM and OM, respectively). In contrast, the morphology of FG did not appear to vary between horizontal and vertical planes, indicating an overall isotropic pore morphology (Figures 3E,F).

### Biomaterial anisotropy and pore size quantification

To be able to relate the cell migration behavior to the individual biomaterial architecture, the pore anisotropy was quantified based on confocal microscopy and second harmonic (SH) imaging.

In MD, most of the material (i.e., the bundles of fiber visible in Figure 3B) had a horizontal orientation between φ = –45° and 45°, although with high variability (Figure 3I). Also in MM, the collagen was distributed predominantly in the horizontal direction (Figure 3J), with a signal intensity 2.6-fold higher along φ = 0° compared to φ = 90°. FG showed an almost isotropic distribution, without any pronounced peaks at specific orientations (Figure 3K). Compared to the other investigated biomaterials, OM showed the highest degree of anisotropy (Figure 3L). Collagen walls were mostly aligned along the vertical direction (φ = 90°), but a smaller peak was identified also in the horizontal direction (φ = 0°), representing the wall-connecting bridges (Figure 3H). Nonetheless, material orientation in the vertical direction was predominant with a signal intensity 7.8-fold higher at φ = 90° compared to φ = 0°.

As a further parameter that is known to influence cell migration, the pore size of the investigated biomaterials was analyzed in the horizontal plane (Figure 3M). In MD, MM, and FG, the distribution curves of the pore diameter peaked below 50 µm (Figure 3N). In FG, individual large pores created additional peaks at values up to 550 µm. In OM, the peak of the pore diameter distribution was between 50 and 100 μm, indicating much larger pores compared to the other three materials. The mean pore size was quantified from the pore size distribution to be 20 ± 8 µm for MD, 27 ± 8 µm for MM, 45 ± 28 µm for FG, and 79 ± 15 µm for OM (Figure 3O). A specific architectural feature observed for MM and OM was the presence of parallel walls oriented along the vertical direction. The distance between these walls was quantified manually as an additional morphological parameter, resulting in wall distances of 79 ± 15 µm for MM and 120 ± 37 µm for OM (Figure 3P), much larger than the detected pore diameter.

### Mechanical characterization

As an additional, indirect approach to characterize the pore architecture, and specifically its anisotropy, the mechanical properties of the investigated biomaterials were measured via monoaxial compression tests. From the obtained stress-strain curves (Supplementary Figure S4), the elastic moduli along the vertical (*E*_*V*_) and horizontal (*E*_*H*_) directions (see Figure 1C) were quantified (Figure 3Q). In general, the compressive elastic moduli of MM, FG, and OM were in the low kPa range. Only MD resulted in higher values of *E*_*H,MD*_ = 90.8 ± 9.8 kPa and *E*_*V,MD*_ = 130.9 ± 29.4 kPa, with a significant difference in elastic modulus between vertical and horizontal directions (*E*_*V,MD*_/*E*_*H,MD*_ = 1.4).

Also the elastic modulus of MM was significantly higher along the vertical (*E*_*V,MM*_ = 4.3 ± 0.7 kPa) compared to the horizontal direction (*E*_*H,MM*_ = 2.6 ± 0.7 kPa), resulting in a ratio of *E*_*V,MM*_/*E*_*H,MM*_ = 1.7. In OM, an even higher difference between the vertical (*E*_*V,OM*_ = 12.3 ± 0.8 kPa) and the horizontal direction (*E*_*H,OM*_ = 2.3 ± 0.7 kPa) was observed, expressed by a high ratio *E*_*V,OM*_/*E*_*H,OM*_ = 5.5. On the contrary and in agreement with the isotropic pore architecture, no significant differences were measured in the compressive elastic modulus of FG in the vertical (*E*_*V,FG*_ = 7.7 ± 1.6 kPa) and horizontal directions (*E*_*H,FG*_ = 8.4 ± 3.0 kPa). Together, the mechanical stiffness data confirmed the dense packing of collagen in MD (indicated by the high compressive elastic modulus), the isotropic pore architecture of FG, and the strong anisotropic properties of OM in the vertical direction. Only for MM, the mechanical properties were surprising. In fact, they indicated an internal architecture with higher elastic modulus in the vertical direction, while the collagen material was found to be distributed primarily along the horizontal direction in the optical evaluation of the structure (Figure 3J).

### Cellular migration distance

The cellular migration into the different biomaterials was evaluated by comparing the proposed novel cell-spheroid-based migration assay (3D spheroid assay, Figure 1B) and a previously established assay that employs a 2D cell layer as cell source (2D layer assay, Figure 1A). The 2D_V_ and 2D_H_ layer assays provide separate information about the migration distance along the vertical (*d*_*2DV*_) and horizontal (*d*_*2DH*_) directions, while the 3D spheroid assay was designed to obtain information on the cellular migration along these two principal directions from a single experiment.

First, the median migration distance from the cell source was quantified using the 3D spheroid assay in comparison to the 2D layer assay (Figures 2A–C). The low thickness of MD prevented a proper placement of the samples in contact with the 2D cell source when testing migration along the horizontal direction. Thus, only a low number of samples was successfully analyzed (n = 3 out of 21), precluding a test of significance for MD in the 2D_H_ layer assay. Contact problems between MD samples and the 2D cell source were observed also in the 2D_V_ layer assay (Supplementary Figure S5). On the contrary, despite an occasional improper adhesion of the spheroid to the material during the initial step, the 3D spheroid assay was successfully performed on MD (n = 8 out of 11 samples successfully analyzed). All other biomaterials were successfully tested with the two different migration assays. The total number of cells detected in each biomaterial and in each type of assay was found to be comparable for MM, FG, and OM, but had a tendency to be lower for MD (Supplementary Figure S6).

**FIGURE 4:**
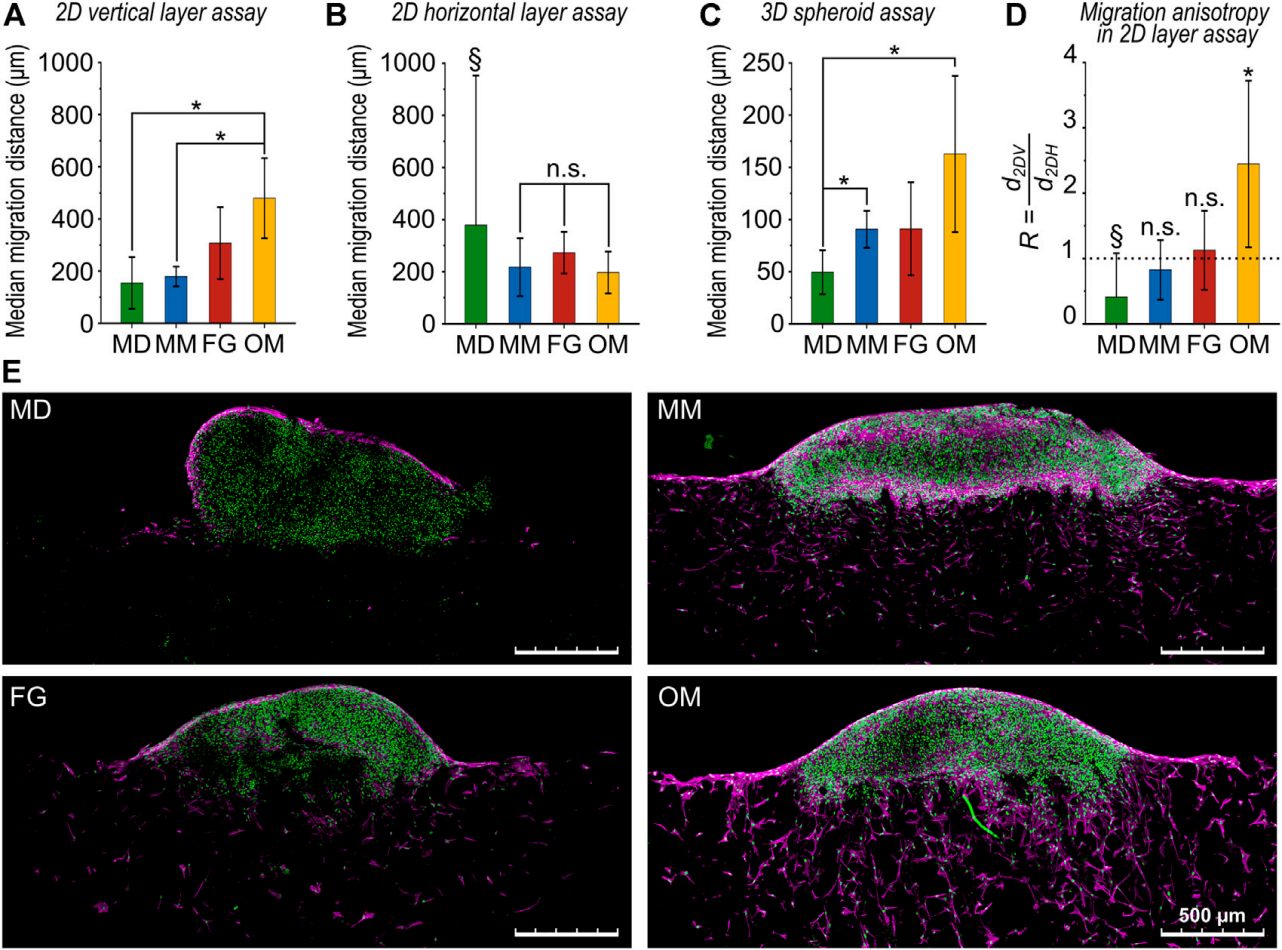
Comparison of cellular migration distances for the investigated biomaterials. **(A)** Migration distance obtained from the 2D vertical layer assay (*d*_*2DV*_), **(B)** the 2D horizontal layer assay (*d*_*2DH*_), and **(C)** the 3D spheroid assay (*d*_*3D*_). All results are given as mean values of the median migration distance ±standard deviation; **(D)** ratio *R* between 2D vertical and horizontal migration distances. The dotted line highlights the value of *R* = 1 indicating isotropic migration behavior. In **(A and C)**, all comparisons between groups that are not indicated as statistically significant (*) were not statistically significant (n.s.). In **(D)**, statistical significance was tested between *d*_*2DV*_ and *d*_*2DH*_ for each biomaterial. Data marked with the § symbol were not statistically tested (for explanation see main text); **(E)** representative images (maximum projections) of the 3D spheroid assay. Cell nuclei are in green and F-actin in magenta. Scale bars are 500 µm.

Based on the 2D layer assay, the median migration distance in the vertical direction increased in the order MD < MM < FG < OM (Figure 4A). Significant differences were found between MD (*d*_*2DV,MD*_ = 154 ± 99 µm), MM (*d*_*2DV,MM*_ = 179 ± 38 µm) and OM (*d*_*2DV,OM*_ = 480 ± 154 µm) while the intermediate value found for FG was not significantly different from the other materials (*d*_*2DV,FG*_ = 307 ± 138 µm). On the contrary, the horizontal 2D layer assay resulted in no significant differences between any of the investigated biomaterials (Figure 4B). For MM, FG, and OM, values of *d*_*2DH,MM*_ = 217 ± 111 μm, *d*_*2DH,FG*_ = 273 ± 80 µm and *d*_*2DH,OM*_ = 196 ± 81 µm were measured, respectively. The value of *d*_*2DH,MD*_ = 378 ± 574 µm for MD is reported here only as an indication due to the above described problems in performing the assay on this material and was not included in the statistical evaluation. The highest median migration distance quantified from the 3D spheroid assay (*d*_*3D*_) was recorded for OM (*d*_*3D,OM*_ = 163 ± 75 µm) and the lowest for MD (*d*_*3D,MD*_ = 49 ± 21 µm) (Figure 4C). MM and FG had comparable median migration distances of *d*_*3D,MM*_ = 91 ± 18 µm and *d*_*3D,FG*_ = 91 ± 45 μm, respectively. Statistically significant differences were detected between MD and MM or OM.

The ratio *R*, calculated as the ratio of the median migration distances in the vertical and the horizontal directions (*R* = *d*_*2DH*_/*d*_*2DV*_) from the 2D layer assays, indicated an isotropic migration behavior with *R* ≈ 1 for both MM and FG (*R*_*MM*_ = 0.8 ± 0.5 and *R*_*FG*_ = 1.1 ± 0.6, respectively) (Figure 4D). The value of *R*_*MD*_ = 0.4 ± 0.7 for MD is here reported only as an indication, as the result is unreliable due to the low number of samples successfully analyzed. In OM, a clear anisotropic behavior with significantly greater migration in the vertical direction was found (*R*_*OM*_ = 2.4 ± 1.3).

### Anisotropy of cellular migration

To evaluate the anisotropy of cellular migration for the 3D spheroid assay, four isodensity migration profiles were calculated for each biomaterial using a semi-automated image analysis routine developed for ImageJ and Matlab (Figures 2D–F). The isodensity profiles outline areas in which cellular density is equal to or higher than a threshold. Specifically, four thresholds were analyzed, identifying regions of cell densities of 50%, 15%, 10%, and 5% of a pre-defined reference cell density (see Materials and Methods section for details). Subsequently, each of these isodensity profiles was fitted with an isodensity ellipse, whose semi-major (*a*) and semi-minor (*b*) axes were considered as measure of migration distance in the horizontal and vertical directions, respectively.

**FIGURE 5:**
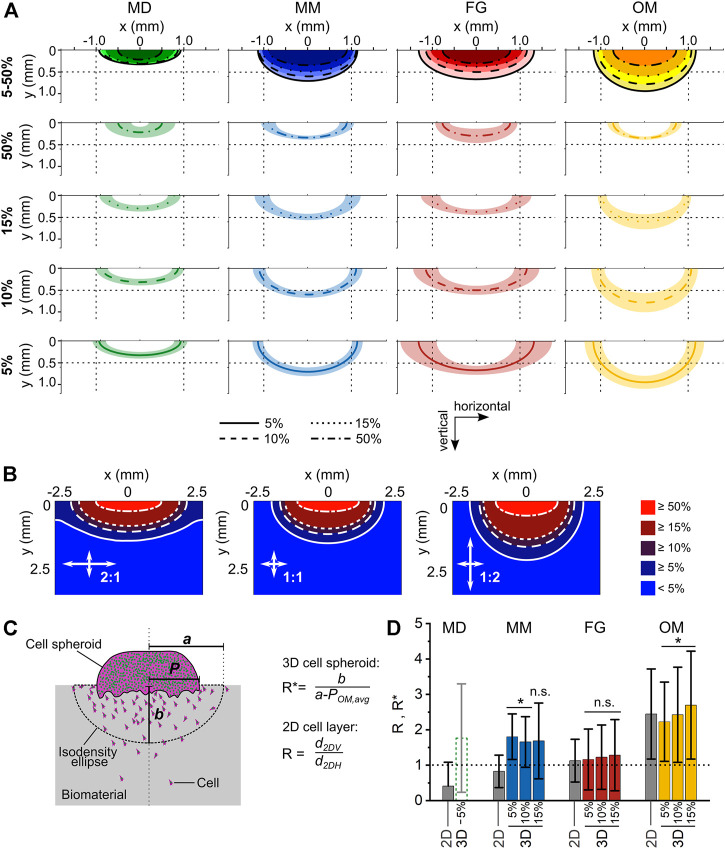
Analysis of anisotropy in cellular migration within the investigated biomaterials. **(A)** Isodensity ellipses for the different biomaterials analyzed at 4 cell density thresholds (5%, 10%, 15%, and 50%). The first row shows the overlay of the mean isodensity ellipses for each biomaterial, while the subsequent rows show the mean values (line) ± standard deviation (shaded area) of each isodensity ellipse. To ease the comparison between the plots, reference lines are shown at 0.5 mm and ±1 mm of the vertical and horizontal coordinates, respectively; **(B)** Diffusion patterns obtained from computational simulations for three ratios of horizontal and vertical diffusion coefficients (2:1, 1:1, and 1:2); **(C)** schematic representation of spheroid radius (*P*), semi-major axis *(a)*, and semi-minor axis *(b)* in the 3D spheroid assay; **(D)** migration anisotropy in 3D spheroid assay as measured with the ratio R* and compared to the ratio *R* obtained in the 2D layer assay (gray bars). Results are given as mean ± standard deviation. The dotted line marks the value of an ideal isotropic migration behavior R* = 1. Statistical significance was tested for the difference between the R* values of the individual biomaterials and R* = 1 and it indicates anisotropic migration.

The least extended migration profile was found for MD (Figure 5A). The horizontal and vertical migration distances calculated for the 5% isodensity ellipse of MD were *a*_*MD,5%*_ = 930 ± 152 µm and *b*_*MD,5%*_ = 327 ± 76 μm, respectively.

For MM, the isodensity plots indicated a more progressive migration into the biomaterial compared to MD, with isodensity ellipses being more separated from each other (Figure 5A). In fact, the horizontal and vertical migration distances calculated for the 5% isodensity ellipse of MM were *a*_*MM,5%*_ = 1,134 ± 124 µm and *b*_*MM,5%*_ = 701 ± 101 μm, respectively. Thus, the vertical migration distance *b* was 2.1-fold higher in MM than in MD.

Amongst the investigated biomaterials, FG had the greatest extension of the migration profile in the horizontal direction. The horizontal and vertical migration distances calculated for the 5% isodensity ellipse of FG were *a*_*FG,5%*_ = 1,317 ± 413 µm and *b*_*FG,5%*_ = 664 ± 103 μm, respectively (Figure 5A). The vertical migration distance *b* in FG was comparable to MM, but 2.0-fold higher than in MD. The average isodensity ellipses in FG were well-separated from each other, indicating a progressive migration even in regions where the cell density was low (5% and 10%). This was in strong contrast to the indistinguishable profiles of MD and even more pronounced than in MM, especially for low density profiles (5, 10, 15% profiles in Figure 5A).

The isodensity ellipses for OM were the most clearly separated ones (Figure 5A) and the material featured a migration profile with the largest extension in the vertical direction. The horizontal and vertical migration distances calculated for the 5% isodensity ellipse were *a*_*OM,5%*_ = 1,167 ± 199 µm and *b*_*OM,5%*_ = 939 ± 150 μm, respectively. Consequently, the vertical migration distance *b* in OM was 1.4-fold higher than in FG, 1.3-fold higher than in MM, and 2.9-fold higher than in MD.

To summarize the observed cell migration profiles, MD showed a barrier-like behavior with flat isodensity profiles, in strong contrast to OM, whose almost semi-circular profiles indicated a preferred migration along the channel-like pores (vertical direction of the biomaterial). The migration profiles of MM and FG showed an intermediate extension between MD and OM, with the isodensity ellipses of FG having a greater horizontal than vertical extension (more similar to MD) and those of MM tending towards the almost semi-circular shape found for OM.

To better interpret the shape of the individual isodensity profiles in respect to the according anisotropy of cell migration, a mass diffusion process with a line source (representing the contact between cell spheroid and biomaterial) was computationally modelled, simulating different ratios between the vertical and horizontal diffusion speed (Figure 5B). An isotropic diffusion process produced semi-elliptic concentration profiles, while anisotropy in the horizontal and vertical directions resulted in semi-elliptic profiles with a greater semi-major axis and semi-circular profiles, respectively. In principle, all experimentally derived migration profiles from the 3D spheroid assays were in between the profiles obtained from the simulation for the 1:2 (preferred horizontal migration/diffusion) and 2:1 (preferred vertical migration/diffusion) situation. For OM, the shape of the isodensity ellipses was found to be in between the 1:1 and the 2:1 diffusion profiles (compare Figures 5A,B), confirming the anisotropic migration behavior imposed by the material. On the other hand, the flat isodensity profiles found for MD matched best the 1:2 profile with preferred horizontal migration/diffusion.

For each biomaterial, the ratio between the 2D_V_ and 2D_H_ migration distances (*R*, see Eq. 3) was defined as the reference value describing the anisotropy of cell migration. As the cell spheroid corresponded to a line source and not to a point source, the definition of an equivalent R* ratio describing the migration anisotropy in the 3D spheroid assay required the subtraction of the radius of the spheroid (*P*) from the semi-major axes *a* of the isodensity ellipses (Figure 5C). As the interface between cell spheroid and biomaterial was growing over time from a small contact during the adhesion phase to an extended interface (>1 mm) at day 5 (Supplementary Figure S7), a universal reference value of *P* was used for all biomaterials, taking into account the dynamic change of the spheroid-biomaterial interface. To determine this value, the spheroid radius *P*_*OM,avg*_ was calculated for the reference material OM to obtain a value *R*_*OM*_ * matching the reference value *R*_*OM*_ from the 2D assays (see Eq. 4). Then, the resulting value *P*_*OM,avg*_ = 745 µm was subtracted from the semi-major axes *a* of the other investigated biomaterials, defining their corresponding R* values according to Eq. 5.

In general, the migration anisotropy calculated for the 3D spheroid assay was only marginally influenced by the cell density value chosen to calculate the isodensity profiles (Figure 5D; Table 1). For MD, whose semi-major axes *a* were of similar value to *P*_*OM,avg*_ for the 10% and 15% isodensity ellipses, the according corrected ratios R* could not be calculated. The ratios R* and *R* were comparable for FG and OM. On the contrary, R* was approximately 2-fold higher than the reference value *R* for MM, indicating a surprising mismatch between the anisotropy of migration derived from the 3D spheroid assay compared to the 2D layer assay.

**TABLE 1:** Anisotropy of cellular migration in the 2D layer and 3D spheroid assays expressed by the ratios *R* and R*, respectively.

Material	*R*	R*_5%_	R*_10%_	R*_15%_
MD	(0.4 ± 0.7)	(1.8 ± 1.5)	—	—
MM	0.8 ± 0.5	1.8 ± 0.6	1.7 ± 0.7	1.7 ± 1.1
FG	1.1 ± 0.6	1.2 ± 0.9	1.2 ± 0.9	1.3 ± 1.0
OM (reference)	2.4 ± 2.3	2.2 ± 1.1	2.4 ± 1.4	2.7 ± 1.5

In R*, the subscript text indicates the corresponding isodensity ellipse (5%, 10% or 15%). The *R* and R*_5%_ value for MD (in brackets) are only reported as an indication.

To exclude that the mismatch between the ratio R* and the reference ratio *R* in MM was the consequence of a potentially different spheroid diameter for MM compared to the other biomaterials, the length of the cell spheroid-material contact (corresponding to 2∙*P*) was measured from images of the 3D spheroid assay (Supplementary Figure S7). As results were comparable for all biomaterials, the approach to use a constant value *P*_*OM,avg*_ = 745 µm to correct for the lateral extension of the cell spheroid proved to be acceptable and allowed the comparison of the migration anisotropy (R* values) between the biomaterials.

### Cellular density

**FIGURE 6:**
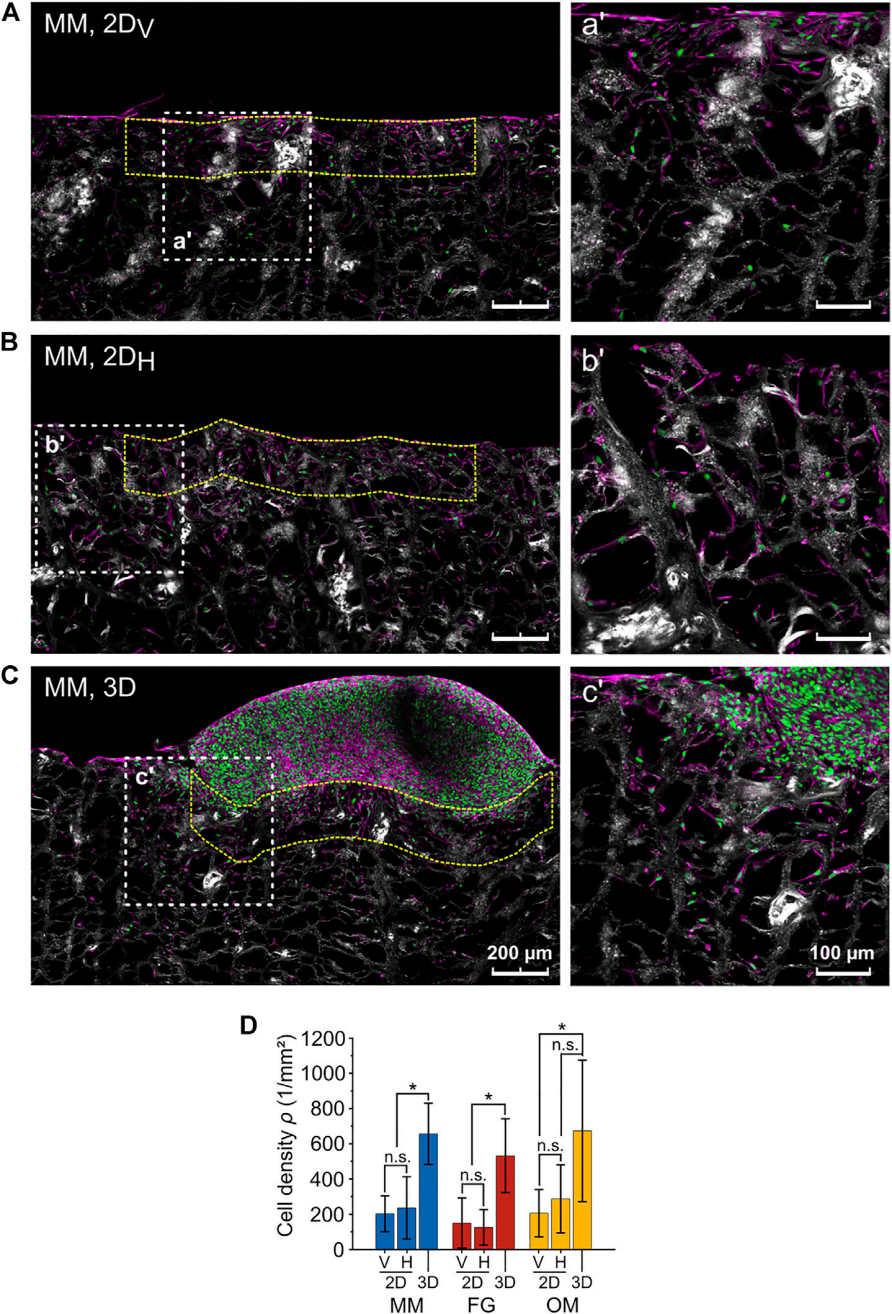
Difference in cellular density between the 2D layer assay and the 3D spheroid assay. **(A–C)** Representative images (maximum projections) of 2D_V_ and 2D_H_ layer and 3D spheroid assays, respectively (MM). Nuclei, F-actin, and collagen are shown in green, magenta, and white (second harmonic signal), respectively. The higher magnification images on the right (a′, b′, c′) show the regions highlighted by the white dashed line squares. The analyzed ROIs are exemplarily marked by dashed yellow lines. Scale bars are 200 µm for overviews and 100 µm for magnified images; **(D)** cell densities as analyzed within the indicated ROIs in the 3D spheroid and 2D_V_ and 2D_H_ layer assays.

The fact that the anisotropy ratios R* and *R* were not matching for MM raised the question on what factor caused the deviation for this particular biomaterial. From the confocal images of the migration assays, a higher cellular density in the 3D spheroid compared to the 2D layer assays was observed. Thus, the cellular density was evaluated in a ROI with height of 200 µm below the contact line with the cell spheroid for the 3D spheroid assay and below the surface of the biomaterial for the 2D layer assay (Figures 6A–C). Cellular densities (*ρ*) did not depend on the orientation of the individual biomaterials in the 2D layer assays, where the following values were measured (Figure 6D): *ρ*_*2DV,MM*_ = 203 ± 102 cells/mm^2^ and *ρ*_*2DH,MM*_ = 236 ± 177 cells/mm^2^ for MM; *ρ*_*2DV,FG*_ = 150 ± 143 cells/mm^2^ and *ρ*_*2DH,FG*_ = 125 ± 101 cells/mm^2^ for FG; and *ρ*_*2DV,OM*_ = 206 ± 134 cells/mm^2^ and *ρ*_*2DH,OM*_ = 287 ± 192 cells/mm^2^ for OM. Measurements in the 3D spheroid assay were consistently higher, reaching values of *ρ*_*3D,MM*_ = 656 ± 174 cells/mm^2^, *ρ*_*3D,FG*_ = 532 ± 209 cells/mm^2^, and *ρ*_*3D,OM*_ = 673 ± 402 cells/mm^2^ in MM, FG, and OM, respectively. Therefore, the initial qualitative observation was supported by the quantitative analysis, confirming that the cellular density at the biomaterial surface in contact with the cell source was significantly higher in 3D spheroid assays compared to 2D layer assays.

## Discussion

A novel cellular migration assay for the pre-clinical characterization of biomaterials for *in situ* Tissue Engineering was developed and used to evaluate three clinically used biomaterials for soft-tissue augmentation, i.e., Mucoderm (MD), Mucomaix (MM), and Fibro-Gide (FG). This study focused on how the migration of cells into the biomaterial is controlled by the pore morphology of the materials (pore size and isotropic/anisotropic architecture) and the according mechanical properties (elastic moduli). The cell-culture substrate Optimaix (OM) served as a well-described reference material (Petersen et al., 2018; Brauer et al., 2019; Herrera et al., 2019; Schreivogel et al., 2019). Compared to established migration assays based on 2D cell sources (Petersen et al., 2018) (2D layer assay, Figure 1A), the 3D spheroid assay (Figure 1B) was designed to improve testing of biomaterials with non-planar surfaces and/or an anisotropic architecture. In fact, the limited extent of the cell source–biomaterial interface in the 3D spheroid assay (point-like cell source) enables the quantitative study not only of migration distances, but also of possible preferential directions of migration.

As the 3D spheroid assay allows the evaluation of cellular migration along two principal directions within one experiment, this method is more time-effective and requires less material compared to a 2D layer assay. All investigated biomaterials were derived from porcine collagen, which minimizes unspecific effects resulting from a different material chemistry. As the biomaterials differ in their mechanical properties and architecture, they represented a good choice to test the here proposed 3D spheroid migration assay regarding its ability to capture differences in cellular migration as a consequence of biomaterial-dependent guidance.

MD is produced from decellularized porcine dermis, which maintains the architecture of the natural extra-cellular matrix (ECM). The other three biomaterials are produced by technical processing of porcine-derived collagen to induce a macroporous morphology. Although the natural architecture of the ECM is lost, the high porosity and the large pore diameters introduced during production were reported to support cell invasion and tissue formation (Thoma et al., 2016; Olde Damink et al., 2018; Petersen et al., 2018). The mechanical and morphological characterizations identified MD and OM as strongly anisotropic biomaterials with preferential orientation in the horizontal and vertical directions, respectively. For MD, the alignment in the horizontal direction matches the structural properties of dermis, from which it derives. For OM, the strong vertical alignment is technically introduced by the manufacturer through directional freezing and freeze-drying of a collagen dispersion, clearly impacting on resulting cell and tissue organization (Petersen et al., 2018; Brauer et al., 2019; Herrera et al., 2019). For MM, the structural properties were not as evidently defined. In fact, the material orientation analysis revealed a preferential material orientation in the horizontal direction (given by the sail-like horizontal brides, Figures 3D,J), while the mechanical characterization showed a higher elastic modulus in the vertical direction (Figure 3Q). A possible explanation is that vertical walls are long, continuous, and mechanically stabilizing elements, while horizontal elements are less consistently connected. Consequently, MM is a material with mixed properties, in which horizontal elements dominate the structural appearance while vertical walls dominate the mechanical properties. Finally, FG was found to be a truly isotropic material from both the structural and the mechanical point of view.

The ratio *R* (see Eq. 3) was introduced in this study to describe the anisotropy of cellular migration as calculated from independent 2D layer migration experiments along vertical and horizontal directions (Figure 4D and Table 1). An anisotropic cellular migration behavior preferentially along the vertical direction was observed in OM (*R*_*OM*_ = 2.4 ± 2.3), which confirms a similar ratio *R* = 2.7 previously reported for OM with primary mesenchymal stromal cells (Petersen et al., 2018). The 2D_V_ and 2D_H_ migration distances in MM and FG indicated an isotropic migration behavior, with values of *R* ≈ 1. For MD, a value of *R* < 1 denoted an anisotropic migration behavior preferentially along the horizontal direction, which is consistent with the membrane-like morphology of the biomaterial and its horizontal fiber orientation. However, the value of *R*_*MD*_ should be interpreted with care due to the low number of successfully performed assays. Interestingly, the results of the 2D_H_ layer assay indicate no significant differences between the migration distances of the investigated biomaterials along the horizontal direction (Figure 4B). In contrast, the clear differences in vertical migration showed that variations of internal material architecture were most pronounced in this direction (Figure 4A). This further underlines the strong limitations of 2D layer-based migration assays and highlights the necessity to perform multiple experiments along the principal biomaterial directions, if those exhibit either a structural or mechanical anisotropy.

The 3D spheroid assay could be successfully performed on all biomaterials, including MD, highlighting the value of the here proposed method. Although a similar trend for the median migration distance from 3D spheroid and the 2D_V_ layer assay was observed, the absolute migration distance was consistently lower in the 3D spheroid assay (Figure 4C). We speculate that this discrepancy is a consequence of the continuous cell supply provided by the spheroid, resulting in a high number of cells with an overall low median migration distance even at a late time point (constant high cell density at the surface of the material). Moreover, the different degree of cell-substrate and cell-cell adhesion in the two types of assays might have played a role. In fact, cells in the 2D layer assay were allowed to adhere to the culture plastic and to each other for only 1 h prior to the placement of the biomaterials, which is the starting point for the cellular invasion process. On the contrary, cells in the densely packed 3D spheroids were pre-cultured for 24 h, providing more time to establish mature cell-cell contacts. Thus, the 2D layer assay can be regarded as representing the invasion of individual cells, while cells in the spheroid have to break away from a dense 3D configuration before entering the biomaterial. This might contribute to the lower migration distance in the 3D spheroid assay compared to the 2D layer assay and it is considered more representative for *in vivo* cell invasion, where cells are recruited from dense adjacent tissues at the site of implantation.

Cell spheroids were occasionally observed to indent into the material and, for certain biomaterials such as MM and OM, to protrude into the pores (Figure 4E). This is considered the consequences of a mechanical pull of the spheroid into the material resulting from collective cellular migration. In fact, collective cellular migration has been previously observed for fibroblasts (Leong et al., 2013; Jiang et al., 2020). Furthermore, the mechanical pull of invading cells might be accompanied by a mechanical push of the spheroid into the biomaterials resulting from the tension generated by the cells at the top surface of the spheroid when adhering to the material (Ehrig et al., 2019). However, the protrusion of the cell spheroid into the biomaterial is not expected to significantly influence the subsequent evaluation of migration anisotropy in the 3D spheroid assay. In fact, the local cellular density used to detect the isodensity profiles was analyzed distant from the cell spheroid–biomaterial interface, especially for the low-density profiles (5% and 10%).

Anisotropy in cellular migration in the 3D spheroid assay was evaluated based on isodensity ellipses (Figure 5A). The isodensity ellipses for the four investigated biomaterials showed a general similarity to the migration profiles predicted by computationally simulating a mass diffusion process from a line source (Figure 5B), indicating that cells explore the 3D material in a density-driven manner as reported before (Leong et al., 2013). It should be mentioned that the computational simulations were here employed only to establish reference profiles of anisotropic migration behaviors, not to describe nor predict the phenomenon of anisotropic cellular migration itself. The representation of the 3D migration environment through 2D *in silico* models was already employed in previous studies to predict cellular migration as a diffusion process (Tortorici et al., 2021). Thus, the approach was considered suitable to illustrate how the migration profiles change in selected situations of material anisotropy.

Concerning the individual materials, the value *R* < 1 (2D assay, Table 1 and Figure 5D) for MD clearly suggested a preferential cell migration along the horizontal direction. Moreover, the comparably flat isodensity ellipses derived from the 3D spheroid assay (Figure 5A) indicated that cells were hindered in proceeding deep into MD, highlighting its barrier-function. However, in contrast to this observation, a value of R*_MD,5%_ > 1 was calculated for MD from the 3D spheroid assay, indicating a preferential cellular migration along the vertical rather than the horizontal direction. This is not only in disagreement with the results of the 2D layer assay, but also with the preferred horizontal orientation of the multi-layered decellularized dermis material (Figures 3A, B). In this context, it must be mentioned that the procedure to calculate anisotropy from the 3D migration profiles is sensitive to variations in cell spheroid attachment that might occur between the different materials. In fact, the length of the cell spheroid radius *P* is subtracted from the lateral extension of the individual profiles (*a*′ = *a* – *P*)*.* In case of material-specific differences in *P*, the here employed approach of subtracting a single reference value for all biomaterials would have been inappropriate. However, *P* was comparable between MD and the other investigated biomaterials (Supplementary Figure S7), suggesting a similar spheroid adhesion behavior. It was rather found that the size of the circular ROIs used to quantify the local cell density (see Materials and Methods section) was inappropriately large for MD, leading to an over-estimation of the depth of cell migration into the material (Supplementary Figure S8). A too large ROI size might also explain the overlap observed for some isodensity profiles in MD (5% and 10% profiles, as well as 15% and 50% profiles). Together, this indicates that for materials with very limited cell invasion (like MD), the ROI size would have to be reduced to increase the spatial resolution of the method. On the other hand, a too small ROI size would lead to a smaller number of cells per ROI and would cause stronger fluctuations in the isodensity profiles for materials that support deep cell invasion and extended profiles. As the goal of this study was to compare different materials, such an adaptation was not performed and a uniform ROI size was applied to all specimens. Consequently, MD was excluded from further quantitative analyses concerning the migration anisotropy.

For FG, the values of R* (from 3D spheroid assay) and *R* (from 2D layer assay) were in good agreement, indicating an isotropic migration behavior with values of R*_FG_ ≈ *R*_*FG*_ ≈ 1. This result was also consistent with the structural and mechanical material characterization, which identified FG as an isotropic biomaterial. Surprisingly, the values of *R* and R* did not coincide for MM. While the 2D layer assay indicated an isotropic migration behavior with *R*_*MM*_ ≈ 1, the 3D spheroid assay resulted in an anisotropic behavior with R*_MM_ ≈ 1.7 (i.e., preferential migration along the vertical direction). Based on the morphological characterization of MM, the parallel vertical walls are regarded as a physical barrier for cellular migration transversally to the direction of their alignment (in this case, the horizontal direction), which would foster an anisotropic cellular migration with *R* and R* > 1. The enhanced structural integrity of the vertical walls compared to the discontinuous structure of the horizontal elements within the pores (Figure 3D) explains the superior vertical cell guidance of the material. This cell-guiding ability is best demonstrated by OM, where only a few thin horizontal elements were present between the vertical walls and, consequentially, a strongly anisotropic migration behavior along the vertical direction was found (*R*_*OM*_ ≈ R*_OM_ ≈ 2.4, see Table 1). We ascribe the mismatch in anisotropy between 2D layer (*R*_*MM*_ = 0.8 ± 0.5) and 3D spheroid assay (R*_MM_ = 1.8 ± 0.6 for the 5% isodensity profile) in MM to the higher cellular density found at the cell source–biomaterial interface in the 3D spheroid assay (Figure 6D). In fact, cell migration in the here investigated assays was clearly cell-density driven. Cells moved from regions of high density (the cell source) into regions with lower density within the biomaterial, a situation resembling autologous cell invasion *in vivo* (Petersen et al., 2018). For MM, the higher total number of cells used in the 3D spheroid assay and the resulting higher cell density in the material might provoke a different interaction of the cells with the vertical and horizontal structural elements of the material compared to the 2D layer assay. The continuous supply with cells from the cell spheroid (3D spheroid assay) keeps the cell density at the material surface high during the entire duration of the experiment, while the density strongly decreases over time in the 2D layer assay as cells distribute within the biomaterial. Following this argumentation, it could be concluded that cells at a higher density are guided more by the vertical walls and hindered less by the horizontal, sail-like elements found between the walls in MM (Figure 3D).

Although a comparison to *in vivo* cellular migration is at this time not available, we propose that the cell-material interaction observed in the 3D spheroid assay and the resulting high cell density are more representative of the *in vivo* cell invasion process than previously used 2D layer assays. In fact, the cell spheroids provide a continuous cell supply over the course of the experiment, which is believed to better model the *in vivo* cell invasion from surrounding tissues compared to the limited total number of cells provided through the 2D layer assay. *In vivo*, cells will simultaneously access the biomaterial from different points of the material surface and might influence each other in the process. Thus, the use of a cell spheroid represents a compromise between the goals of offering a high number of cells and ensuring a continuous supply on the one hand, and the spatial restriction in cell-material contact that is necessary to quantify spatial migration profiles on the other hand. However, based on the analyzed spatial migration properties obtained in for the individual materials in this study, predictions could be made on the 3D *in vivo* cell recruitment (e.g., by computational simulations) if the cell availability at each point of the material surface would be known. In the end, however, a comparison with *in vivo* experiments will be required to prove this interpretation. In this context, a broader application of the here-suggested method will help to further evaluate its suitability to predict the *in vivo* cell invasion into different kinds of biomaterials. It should be further mentioned that biomaterials with a more complex anisotropy, e.g., showing distinct architectural features in more than two spatial directions, would require additional cutting planes for the characterization of the 3D migration behavior. In this case, one might adapt the approach presented here to image the entire population of migrated cells by optical clearing and full 3D microscopy (e.g., using light sheet microscopy) and to extend the analysis shown in Figure 5 towards 3D isodensity profiles. However, for the dense, strongly light-scattering collagen scaffolds used in this study and the large spatial extension of the migration profile in these materials, such an approach has not shown to be feasible.

In conclusion, we established an easy-to-use *in vitro* assay to evaluate cellular migration within biomaterials that can be performed with standard cell culture equipment. The approach proposed here was shown to be a suitable method for pre-clinical testing of biomaterials with different architectures. Placing a 3D cell spheroid directly upon the biomaterial was proven to successfully overcome issues related to the initial cell–biomaterial contact as observed using 2D cell sources. In fact, the 3D spheroid migration assay enables an application on biomaterials whose shape or surface topography made them unsuitable for existing assays that require entirely planar material surfaces. Moreover, the novel 3D spheroid assay enables the evaluation of preferential directions of migration (anisotropy) in a single experiment and is expected to better represent the *in vivo* situation, as cells can continuously be recruited from the cell source (3D spheroid) into the biomaterial. The resulting higher cell density is proposed to have an influence on cellular motility. Thus, the approach is expected to provide extended information concerning the ability of individual biomaterial types to support cell invasion for biomaterial-assisted tissue healing or regeneration.

## Data Availability

The original contributions presented in the study are included in the article/Supplementary Material, further inquiries can be directed to the corresponding author.
