# Supplementary material for: Characterizing cell recruitment into isotropic and anisotropic biomaterials by quantification of spatial density gradients in vitro
Source: Front Bioeng Biotechnol. 2022 Aug 5;10:939713. doi: 10.3389/fbioe.2022.939713 (PMC9389461; doi:10.3389/fbioe.2022.939713)
Supplement: Supplementary file 1 [file DataSheet1.docx]

Supplementary Material


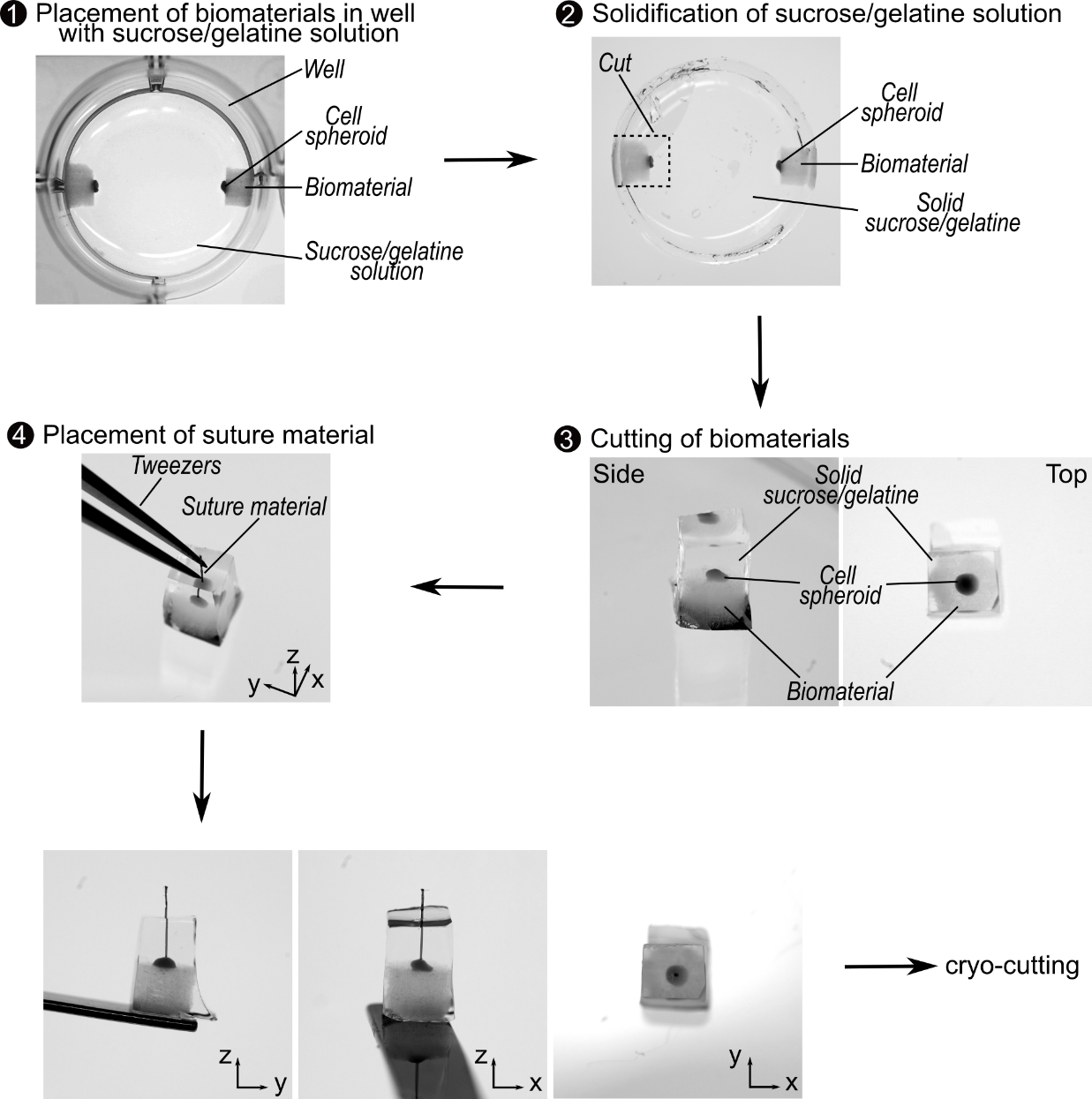


**Supplementary Figure 1.** Schematic representation of sample processing for the 3D spheroid assay. 1) Samples are placed in 12-well plates (up to three equally-spaced samples per well) and the sucrose/gelatine solution is added, avoiding floating of the samples; 2-3) after the solidification of the sucrose/gelatine solution (1h incubation at 4°C), the block is removed from the well and individual samples are cut out; 4) the center of the cell spheroid is identified by sticking a piece of suture material in the solid sucrose/gelatine layer on top of the spheroid to indicate the end-plane for cryo-cutting.


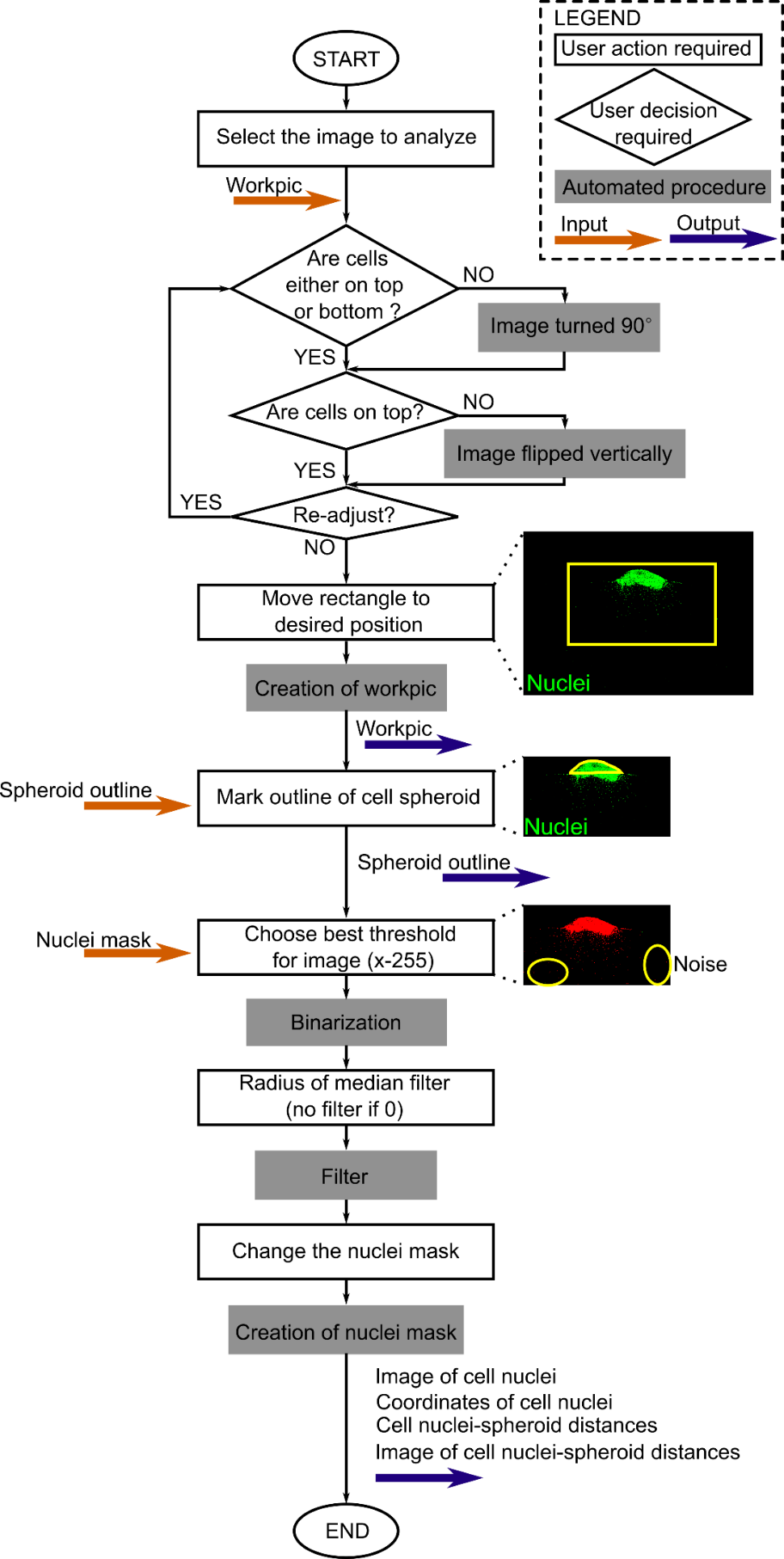


**Supplementary Figure 2.** Workflow of the analysis of the cellular migration distance based on the developed ImageJ macro. If the macro is applied multiple times to the same image, some of the previously defined items (here indicated as input) can be either employed or overwritten. The results of the procedure are indicated as output. In choosing the threshold, priority should be given to the proper identification of cell nuclei, as noise (for example deriving from the biomaterial) can be manually excluded.


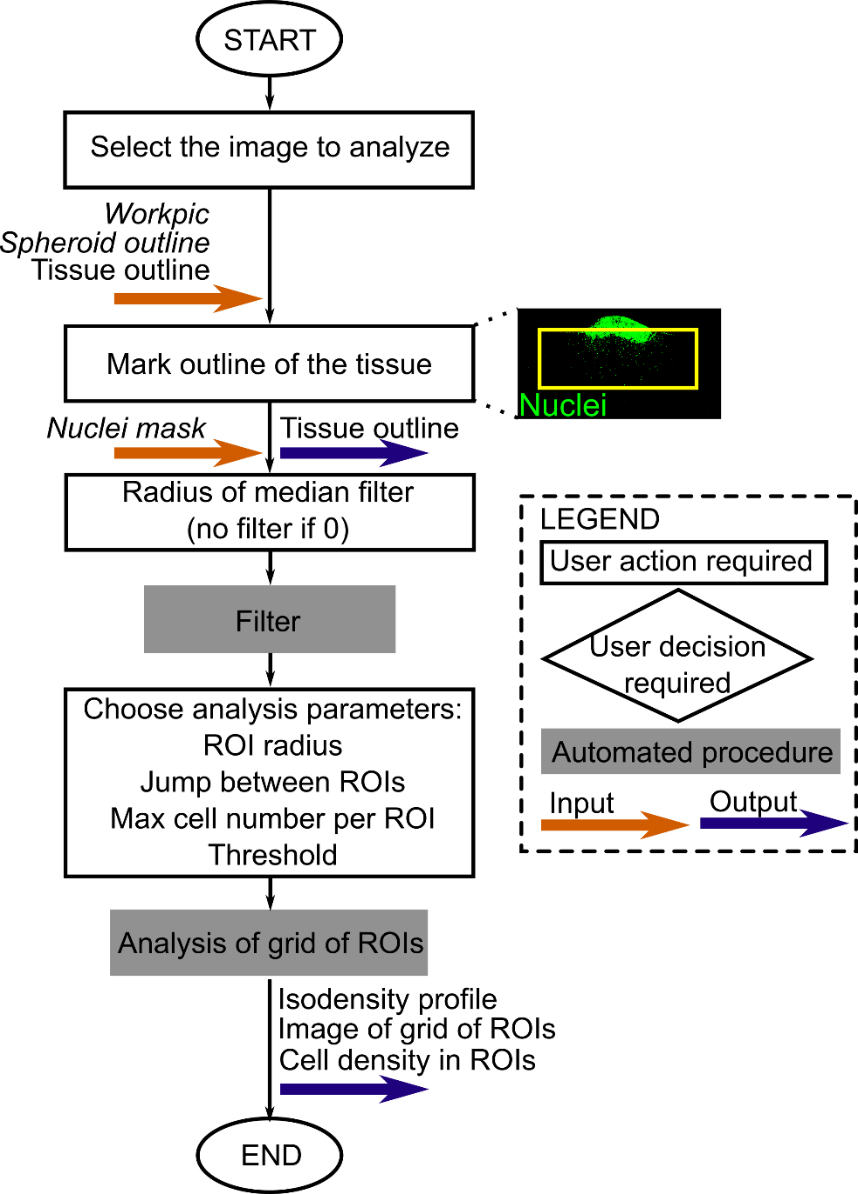


**Supplementary Figure 3.** Workflow of the analysis of the isodensity profiles based on the developed ImageJ macro. If the macro is applied multiple times to the same image, some of the previously defined items (here indicated as input) can be either employed or overwritten. Input items written in italics derive from the macro for the analysis of migration distance. The results of the procedure are indicated as output.


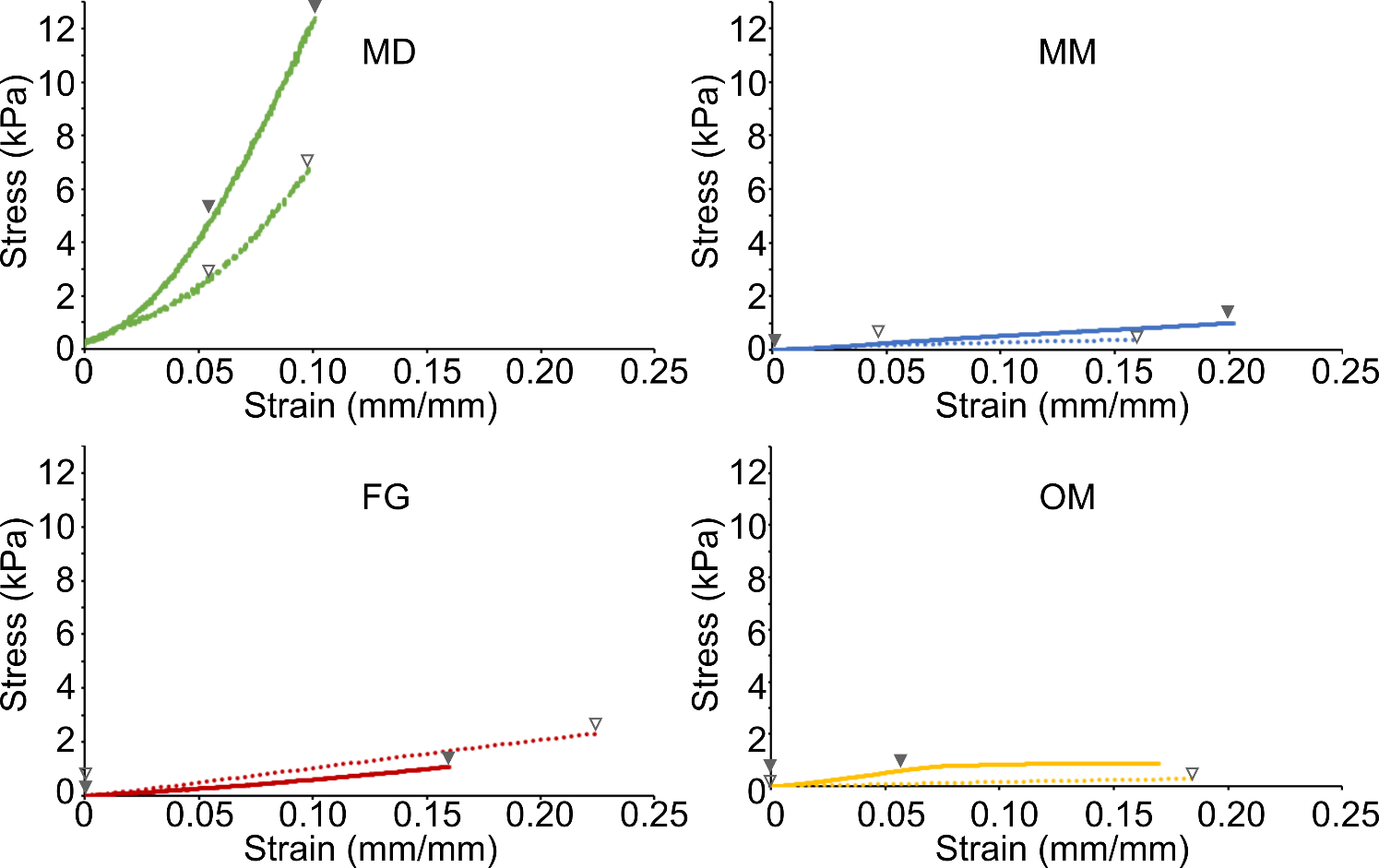


**Supplementary Figure 4.** Representative stress–strain curves of the investigated biomaterials. Solid and dotted lines represent samples tested in compression along the vertical and horizontal directions, respectively. The beginning and the end of the linear traits on which the elastic modulus was evaluated is marked by arrowheads. Specifically, full and empty arrowheads mark the samples tested along the vertical and horizontal directions, respectively.


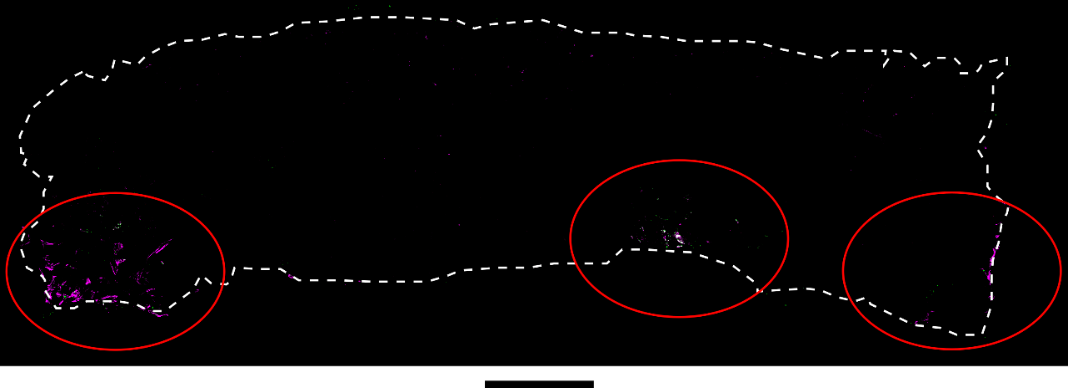


**Supplementary Figure 5.** Representative confocal image (maximum projection) of 2D vertical layer assay in a MD sample. Cell nuclei and F-actin are visualized in green and magenta, respectively. The white dashed line outlines the biomaterial, while the red circles highlight regions of the sample populated by cells. The non-planar surface of the biomaterial resulted in the invasion of a low number of cells. The scale bar is 500 µm.


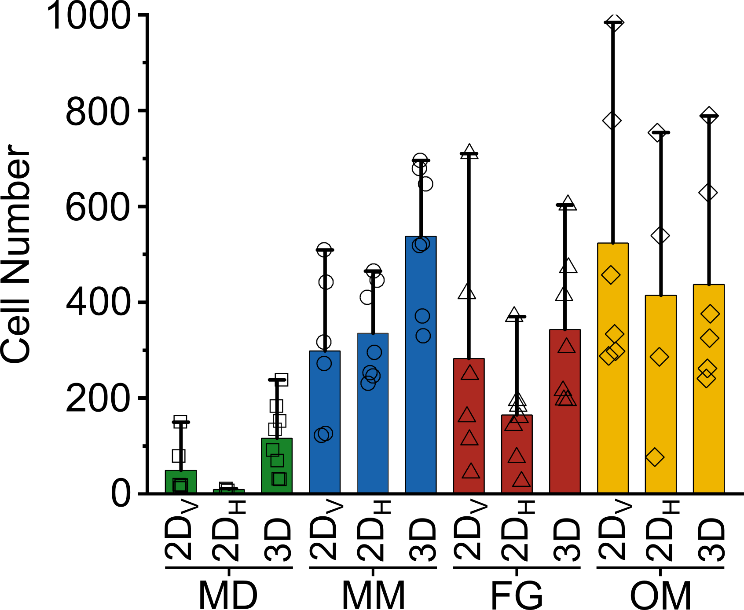


**Supplementary Figure 6.** Number of cells detected in the confocal images used for the analysis of the migration distance in 3D spheroid and 2D layer assays. Each symbol represents one analysed sample. The height of the columns is the mean value and the error bars show the data range within 1.5*Interquartile range.


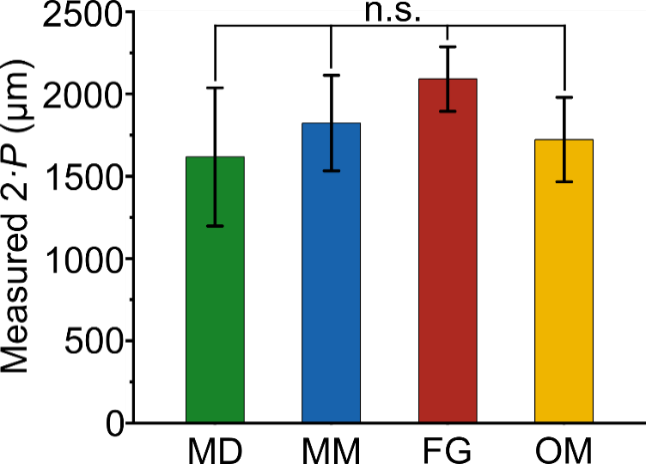


**Supplementary Figure 7.** Length of the cell spheroid-material contact manually measured from images of the 3D spheroid assay corresponding to two times the cell spheroid radius (2∙*P*).


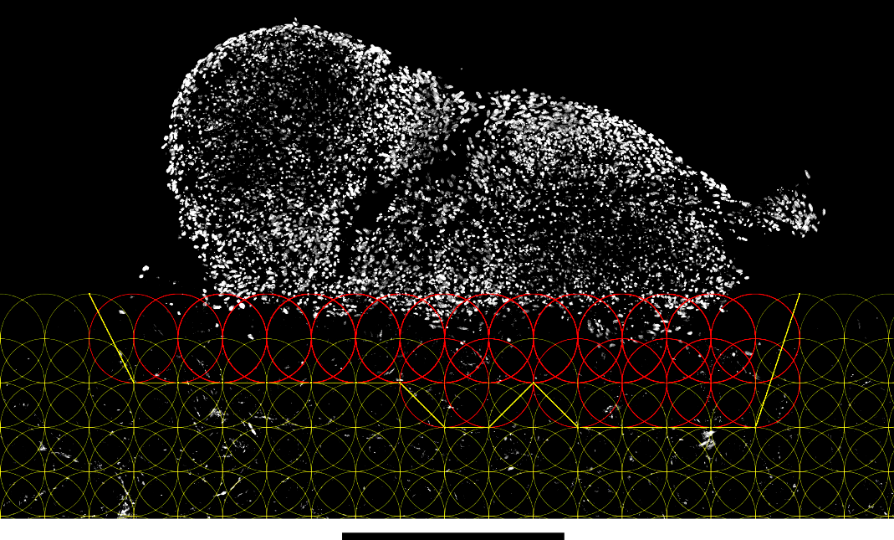


**Supplementary Figure 8.** Representative image of a MD sample during the analysis of the 3D spheroid assay. The yellow segmented line shows the 5% isodensity profile. Although the cell nuclei (in gray) are found mostly on the surface of the biomaterial, the large circular ROI employed for the analysis (diameter of 200 µm) results in the positioning of the points of the polygonal line in the bulk of the biomaterial, thereby overestimating the extent of migration along the vertical direction. The scale bar is 500 µm.
